# Adjoint-based PDE-constrained optimization of viscoelastic floating membrane for maximum wave power absorption

**DOI:** 10.1007/s00158-026-04270-5

**Published:** 2026-02-26

**Authors:** Kareem El Sayed, Shagun Agarwal, Andrei Metrikine, Oriol Colomés

**Affiliations:** Faculty of Civil Engineering and Geosciences, Delft University of Technology, Stevinweg 1, 2628 Delft, CN The Netherlands

**Keywords:** Floating membrane, Floating breakwater, Flexible wave energy converter, Hydroelasticity, Finite element method, PDE-constrained optimization

## Abstract

Viscoelastic floating membranes can be used as flexible wave breakers to protect coastal and offshore structures or as flexible wave energy converters. Despite their potential, the role of viscoelastic floating membranes in optimally harvesting or dissipating wave energy remains largely unexplored, particularly regarding how spatially varying material properties influence their performance. To address this gap, we develop an adjoint-based PDE-constrained optimization framework, built on a monolithic finite element formulation of the coupled fluid–structure interaction problem, to investigate and optimize the viscoelastic properties of floating membranes. This methodology enables a systematic optimization of design parameters such as the mass, tension, and damping, which govern the response of the membrane at different wave conditions. In this study we demonstrate that the proposed methodology allows for the optimization of homogeneous and inhomogeneous properties of membranes for different wave excitation frequencies, leading to significant improvements in energy absorption. The framework is implemented in Julia using the Gridap package ecosystem, which enables automatic differentiation of adjoints and avoids the need to derive complex adjoint formulations.

## Introduction

The recognition of wave energy as an energy-dense renewable resource is well established. Estimates suggest that the global capacity for wave energy could reach approximately 2 TW (Drew et al. 2009). Amidst a growing global focus on sustainable energy solutions, numerous countries are keen to transition from fossil fuels to renewable sources, positioning wave energy as a promising alternative. Research points to multiple advantages associated with the harvesting of energy from sea waves, which is considered more reliable and capable of generating power 90% of the time (López et al. 2013). The three primary types of wave energy converters (WECs) identified are attenuators, point absorbers, and terminators (Drew et al. 2009). Various studies are available on the optimization of these three types of WECs (Shadmani et al. 2023; Wang and Wang 2023; Zhang et al. 2021), consisting mostly on bulky rigid and possibly articulated structures. In addition, new concepts are appearing in the literature, such as flexible WEC (Michele et al. 2020; Collins et al. 2021; Boren 2021). In this work we focus on the structural optimization of floating viscoelastic membranes, which qualify as a flexible body WEC. This type of WEC is notably more efficient in harvesting wave energy than its rigid counterparts (Li and Xiao 2022). Designed as deformable structures, flexible body WECs, such as those using dielectric materials, are adept at converting wave energy into mechanical energy through the interaction of the wave surface with the membrane. This wave–structure interaction induces a strain field in the membrane, enabling the harnessing of wave energy via, for example, the deformation of the dielectric material. Additionally, the deployment of such flexible membranes, regarded as floating structures, provides the added benefit of reducing wave loads on offshore infrastructure (Holkema et al. 2023). This can lead to the use of floating viscoelastic membranes as flexible wave energy harvesters or flexible wave breakers.

Unlike regular wave energy converters, which cover smaller surfaces and require deployment in large arrays to be effective (Shadmani et al. 2023), flexible WECs, such as viscoelastic membranes, offer significant advantages because of their expansive size and, theoretically, infinite degrees of freedom. This configuration enables them to capture substantial amounts of wave energy over extensive areas. Additionally, the considerable number of degrees of freedom present in these membranes allows for enhanced optimization. Specifically, the properties of a viscoelastic membrane can be treated as inhomogeneous properties throughout its span. By fine-tuning these inhomogeneous properties, it is possible to broaden the bandwidth of the harvested wave energy, thus optimizing energy capture. This contrasts with regular WECs, which typically exhibit a narrow resonance bandwidth dictated by their fixed geometry. It is important to acknowledge that rigid-body WECs often employ active control strategies such as latching to improve the wave energy capture performance (Qin et al. 2025). Applying such high-frequency control to flexible membranes is challenging due to their distributed mass and infinite degrees of freedom. Instead, viscoelastic membranes are suited for adaptive passive control or slow-tuning, where properties are adjusted to match the dominant frequency of the wave climate via, for example, dynamically adjusted mass distribution. In this context, the proposed optimization framework is particularly valuable. However control strategies of such type of structures is a topic out of the scope of this work and could be object of a follow-up study.

The modeling of floating viscoelastic membranes, whether as wave energy converters or as floating breakwaters, results in the same mathematical formulation. Consequently, our study addresses the harvest and dissipation of energy by floating viscoelastic membranes in its broadest sense. Here we base the optimization framework on a monolithic finite element formulation for the wave–structure interaction problem presented in recent works (Agarwal et al. 2024; Colomés et al. 2023). The formulation introduced in the cited studies couples linearized potential flow and floating structure equations, e.g., viscoelastic membrane, for modeling arbitrarily shaped floating structures in varying sea-bed topography. The energy dissipation or harvesting mechanism of floating viscoelastic membranes considered in this work comes from the addition of a structural viscous damping term, as has previously been done in other studies (Sree et al. 2021; Trivedi and Koley 2022). Additionally, the proposed formulation allows the optimization process to be applicable to very large floating structures (VLFS) in general (Koley et al. 2022). Note that in studies similar to ours, the absorption of wave energy through viscous damping has been successfully integrated into the formulation (Koley et al. 2022). It is also worthwhile noting that in the previous works (Colomés et al. 2023; Agarwal et al. 2024), the material properties are assumed to be homogeneous across the structure, while in this paper we also consider spatially varying material properties.

To maximize wave power absorption or dissipation, the material properties of the membrane must be tuned to align its natural frequencies with the excitation frequency. Optimization is therefore necessary to navigate the vast design space of inhomogeneous properties and identify the specific configurations that maximize this resonant interaction. The design of viscoelastic membranes with spatially varying properties results in a high-dimensional structural optimization problem, where mass distribution, membrane tension, and structural viscous damping must be optimized to maximize energy absorption or dissipation. This leads to a partial differential equation (PDE)-constrained optimization problem, where the governing hydroelastic equations define the feasible design space. One of the main challenges when the design variables are distributed functions, in that case over the membrane, is that the number of optimization parameters becomes prohibitively large for classical gradient-free methods. To address this challenge, we adopt an adjoint-based optimization approach, see e.g., (Solano et al. 2022; Lu et al. 2025). The adjoint method enables efficient computation of gradients of the objective function with respect to the design parameters, making the optimization problem more efficient. The adjoint formulation is embedded in the monolithic finite element framework introduced in Agarwal et al. (2024), ensuring a stable and accurate treatment of the two-way fluid–structure interaction for a wide range of structural properties. Furthermore, an additional degree of novelty of the proposed work is the implementation of the structural optimization framework in the Julia programming language using the Gridap.jl package (Verdugo and Badia 2021), leveraging automatic differentiation to obtain adjoints directly from the discrete equations. This avoids the need to manually derive complex adjoint formulations, significantly reducing development time and minimizing potential sources of error.

Although significant progress has been made in studying flexible floating structures, the existing literature often relies on simplifying assumptions, such as trivial bathymetry, homogeneous material properties, and basic geometries, which limit their applicability to real-world scenarios. For example, Zhang and Schreier (2022) focus on the hydroelastic behavior of very flexible floating structures, but do not address the optimization of material properties or complex geometries. Similarly, Khabakhpasheva and Korobkin (2002) analyze the dynamics of floating beams in simplified 2D configurations, while Meylan et al. (2017) investigate porous elastic plates, emphasizing wave scattering but neglecting non-trivial bathymetry and inhomogeneous material properties. In reference Renzi (2016), on the other hand, focuses on the coupling between piezoelectric electrical components and flexible floating structure models, offering valuable insights into energy conversion, but not studying the optimized membrane properties. Moreover, because of these simplifications, researchers are often able to model the considered problem using analytical solutions. These studies, while insightful, do not address the challenges posed by arbitrary 2D membrane shapes in 3D fluid environments or the systematic optimization of inhomogeneous viscoelastic properties for energy harvesting and dissipation. This work provides a new general numerical formulation that enables the assessment of floating flexible viscoelastic membranes with inhomogeneous properties and arbitrary shape. While this study focuses on standard geometries to isolate the effects of material properties, the underlying Finite Element framework is inherently capable of modeling membranes of complex or irregular geometry, see (Colomés et al. 2023). This flexibility is essential for designing platforms that must integrate with specific offshore infrastructures or conform to irregular coastal features. This formulation is supplemented with a gradient-enabled optimization approach through the use of adjoints, which allows efficient optimization of non-constant material properties for optimal wave energy harvesting and dissipation. Furthermore, the proposed methodology is inherently scalable to large 3D problems, with non-trivial bathymetry, and allowing inhomogeneous membrane properties. The effectiveness of the proposed approach will be demonstrated later in the paper through a real-world scenario, utilizing a 3D model that incorporates complex bathymetry and membrane geometry, while accounting for inhomogeneous membrane properties.

In this work, we introduce an adjoint-based PDE-constrained optimization framework for floating viscoelastic membranes with several novel aspects, namely 1) we demonstrate that the proposed framework enables the optimization of both homogeneous and spatially varying material properties; 2) we employ an adjoint-based optimization method within an unconditionally stable monolithic finite element framework, allowing efficient gradient computation in high-dimensional design spaces arising from wave–structure interaction problems; and 3) the framework is general, enabling its scalability to arbitrary geometries, realistic bathymetry, and very large floating structures.

This paper is structured as follows: In Sect. 2, the problem is defined by introducing the governing equations. In Sect. 3, the numerical formulation of the problem is defined, including the formulation for the design parameters that we opt to optimize. In Sect. 4, the entire optimization derivation can be found, which is split into two main parts: the definition of the objective function and the derivation of the adjoint-gradient. In Sect. 5, we present the results obtained and the corresponding discussion of the results. Finally, we conclude the research in Sect. 6.

## Problem setting

The definition of the problem can be split into two parts. In the first part of the problem definition, we briefly discuss and describe the theory used to model the membrane. This model is derived from Colomés et al. (2023) and Agarwal et al. (2024). However, this paper is mainly written to emphasize the optimization of viscoelastic membranes so that we can maximize energy absorption. Hence, the second part will be mainly focused on the derivation of the objective function.

**Fig. 1:**
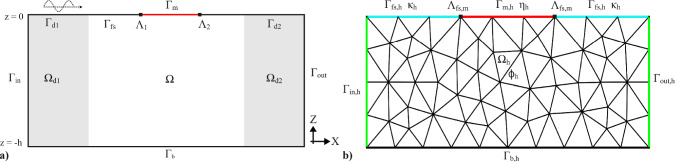
Schematic overview of the numerical setup. (a) The computational domain $\Omega$ including the inlet $\Gamma _{in}$, outlet $\Gamma _{out}$, damping zones $\Omega _{d1,d2}$, and the floating membrane $\Gamma _{m}$. (b) The finite element discretization showing the mesh $\Omega _{h}$ and the degrees of freedom on the membrane and free surface (Agarwal et al. 2024)

The viscoelastic membrane is modeled as a 1D membrane on the surface of a 2D fluid domain $\Omega$. This approximation can be used in the design of large membranes, whose lateral dimension (perpendicular to the wave direction) is much larger than the in-line dimension. This fluid domain is bounded by an inlet surface $\Gamma _{in}$, bottom $\Gamma _{b}$ and an outlet surface $\Gamma _{out}$. Furthermore, it is bounded by the free surface $\Gamma _{fs}$, which is split into three zones: two damping zones, $\Gamma _{d1}$ and $\Gamma _{d2}$, and the remaining undamped region. Finally, the domain is bounded by the floating 1D membrane denoted as $\Gamma _{m}$ which has left boundary points $\Lambda _1$ and $\Lambda _2$. Figure 1 shows the setup of the domain used. Before defining any of the governing equations of the problem, some assumptions are introduced in the model.

- **Assumption 1** We consider the flow within $\Gamma$ to be incompressible, inviscid, and irrotational. Furthermore, the fluid domain is described using linearized potential flow theory, which means that the free surface elevation is assumed to be small compared to the wave length and the water depth.
- **Assumption 2** There is no air gap between the free surface of the fluid and the floating membrane.
- **Assumption 3** The membrane is a thin homogeneous membrane with small transverse deformation and we assume that there is no significant surge displacement.

The limitation of these assumptions is that the results are primarily valid for small steepness waves. This modeling choice is standard in offshore hydrodynamics, as it enables efficient frequency-domain analysis and has been verified in earlier studies (Colomés et al. 2023; Agarwal et al. 2024) for a wide range of linear scenarios.However, situations where the membrane and free surface separate require a nonlinear time-domain formulation due to additional physical processes such as air entrapment and wave overtopping. These nonlinear effects are outside the scope of the present work and will be investigated in future work by building on the insights from the current linearized analysis.

The governing equations are formulated using three variables: 1) the scalar velocity potential $\phi$ defined throughout the fluid domain $\Omega$, 2) the elevation of the free surface $\kappa$ defined along $\Gamma _{fs}$, and 3) the transverse membrane deflection $\eta$ defined along $\Gamma _m$.

### Linear potential flow theory

Under the prescribed assumptions there exists a scalar potential field $\phi$ such that $\textbf{u} = \nabla \phi$, where $\textbf{u}$ is the velocity vector in the fluid domain. Since we consider our flow to be incompressible, we know that $\nabla \cdot \textbf{u} = 0$, which gives the following governing equation:

1 $$\begin{aligned} \Delta \phi = 0 \quad \text {in} \quad \Omega . \end{aligned}$$

For the bottom boundary, no flow of water can occur through the bottom. The inlet and outlet boundaries are controlled in the sense that there will be corresponding uni-directional flow of the water through these boundaries. This results in the following boundary conditions:

2 $$\begin{aligned} \vec {n} \cdot \nabla \phi&= 0&\text {on} \quad \Gamma _b , \end{aligned}$$

3 $$\begin{aligned} \vec {n} \cdot \nabla \phi&= u_{in}&\text {on} \quad \Gamma _{in} , \end{aligned}$$

4 $$\begin{aligned} \vec {n} \cdot \nabla \phi&= u_{out}&\text {on} \quad \Gamma _{out} . \end{aligned}$$

We can express the pressure at the free surface $\Gamma _{fs}$ using the linearized Bernoulli equation. In (5), $\rho$ represents the fluid density and *g* represents the gravitational acceleration.

5 $$\begin{aligned} p = -\rho \phi _{t} - \rho g \kappa . \end{aligned}$$

Since the free surface is exposed to the atmosphere, the gauge pressure at this interface is zero. This results in the following dynamic condition at $\Gamma _{fs}$:

6 $$\begin{aligned} \rho \phi _{t} + \rho g \kappa = 0 \quad \text {on} \quad \Gamma _{fs} . \end{aligned}$$

In order to apply this dynamic condition in the potential flow problem, we utilized the kinematic free surface boundary condition, which states that the free surface $\kappa$ moves with the vertical flow velocity at the free surface. This gives us

7 $$\begin{aligned} \vec {n} \cdot \nabla \phi = \frac{\partial \kappa }{\partial t} = \kappa _{t} \quad \text {on} \quad \Gamma _{fs} . \end{aligned}$$

### Viscoelastic membrane in 1D

The 1D membrane (8) describes the deformation of a thin, homogeneous, and inextensible membrane subjected to transverse pressure, denoted as $p_m$, where material damping is taken into account. The membrane has a density $\rho _{m}$ and thickness $h_{m}$, which, when combined, gives us the mass per unit area defined as $m = \rho _{m} h_{m}$. Furthermore, in (8), we defined a uniform pre-tension *T* and the viscosity of the membrane as $\tau$.

8 $$\begin{aligned} p_m = m \eta _{tt} - T \eta _{xx} - T\tau \eta _{xxt} . \end{aligned}$$

(8) has two essential elements in our model: the restoring force $T\eta _{xx}$, which resembles a spring, and the damping force $T\tau \eta _{xxt}$, which resembles a dashpot. This approach is based on the Voigt model, where the spring and dashpot are arranged in parallel (Sree et al. 2021; Trivedi and Koley 2022). Note that (8) assumes homogeneous properties, however, the formulation described in this work can also be applied to inhomogeneous material properties, i.e., *m*(*x*) and or $\tau (x)$, see Sect. 5.5. To analyze an undamped system, we set $\tau = 0$ to eliminate the damping term in the governing equation of the membrane. Furthermore, for boundary conditions on the membrane edges, we considered two possible scenarios. The edges are fixed and free boundary conditions, defined as

9 $$\begin{aligned} \eta&= 0&\text {fixed edge} , \end{aligned}$$

10 $$\begin{aligned} \eta _x&= 0&\text {free edge} . \end{aligned}$$

### Coupling boundary condition

Note that we restricted this work to 1D structures, but it can be directly generalized to 3D cases (Agarwal et al. 2024). Having defined the governing equation of the 1D membrane, we can express the boundary condition between the free surface of the fluid and the membrane. According to Assumption 2, there is no air gap between the fluid and the membrane. This means that the free surface velocity under the membrane should be equal to the transverse interface of the membrane itself, resulting in a kinematic condition at the fluid–membrane interface. For the dynamic interface condition, the pressure on the water surface, defined in (5), should be equal to the pressure exerted by the membrane. Thus, we obtain the following two boundary conditions:

11 $$\begin{aligned} \vec {n} \cdot \nabla \phi&= \eta _{t}&\text {on} \quad \Gamma _m , \end{aligned}$$

12 $$\begin{aligned} m_{\rho }\eta _{tt} - T_{\rho }\eta _{xx} - T_{\rho } \tau \eta _{xxt} + \phi _t + g\eta&= 0&\text {on} \quad \Gamma _m . \end{aligned}$$

Here, $m_{\rho } = m/\rho$ is the submerged draft of the membrane, while $T_{\rho } = T/\rho$. Having defined the entire problem in the time domain, we can now focus on the parameters that will be essential for optimization. However, we first describe the problem in the frequency domain.

### Frequency-domain analysis

Since we aim to optimize the floating membrane, it is more convenient to define the problem in the frequency domain. The frequency domain allows us to perform the optimization analysis per wave frequency. This is convenient, especially when performing optimization, where the method/algorithm used may employ a recursive workflow. Since the described model is a linear model, we may assume that $\phi (x,z,t)=\overline{\phi }(x,z)e^{-i\omega t}$, $\eta (x,t)=\overline{\eta }(x)e^{-i\omega t}$, and $\kappa (x,t)=\overline{\kappa }(x)e^{-i\omega t}$. Importantly, we are dealing with complex-valued functions $\overline{\phi }(x,z)$, $\overline{\eta }(x)$, and $\overline{\kappa }(x)$. By substituting these relations into the governing equations, we obtain the frequency-domain formulation of the problem.

13 $$\begin{aligned} \Delta \overline{\phi }&= 0&\text {in} \quad \Omega , \end{aligned}$$

14 $$\begin{aligned} \overline{\phi }_z + i \omega \overline{\kappa }&= 0&\text {on} \quad \Gamma _{fs} , \end{aligned}$$

15 $$\begin{aligned} \overline{\phi }_z + i \omega \overline{\eta }&= 0&\text {on} \quad \Gamma _{m} , \end{aligned}$$

16 $$\begin{aligned} -i \omega \rho \overline{\phi } + \rho g \overline{\kappa }&= 0&\text {on} \quad \Gamma _{fs} , \end{aligned}$$

17 $$\begin{aligned} -m_{\rho }\omega ^2\overline{\eta } - T_{\rho }(1-i \omega \tau )\overline{\eta }_{xx} - i \omega \overline{\phi } + g\overline{\eta }&= 0&\text {on} \quad \Gamma _{m} , \end{aligned}$$

18 $$\begin{aligned} \vec {n}\cdot \nabla \overline{\phi }&= 0&\text {on} \quad \Gamma _{b} , \end{aligned}$$

19 $$\begin{aligned} \vec {n}\cdot \nabla \overline{\phi }&= u_{in}&\text {on} \quad \Gamma _{in} , \end{aligned}$$

20 $$\begin{aligned} \vec {n}\cdot \nabla \overline{\phi }&= u_{out}&\text {on} \quad \Gamma _{out} . \end{aligned}$$

Throughout this study, the optimization results are analyzed in the context of the *wet modes* of the system. We denote by *dry modes* the eigenmodes that describe the vibration of the membrane in a vacuum, based solely on its mass and stiffness. The *wet modes* of a floating structure represent the eigenmodes of the fully coupled fluid–structure system. These modes account for the added mass effect of the surrounding fluid, which lowers the natural frequencies, and the hydrostatic-gravitational stiffness provided by the surrounding fluid. As shown in previous work (Agarwal et al. 2024), the resonance of the floating membrane and thus its maximum power absorption occurs when the excitation frequency aligns with these wet natural frequencies, that is $\omega = \omega _{n}^{w}$, rather than the dry natural frequencies.

## Numerical formulation

### Monolithic weak form

Once we have the final problem in the frequency domain as defined in (13)-(20), we proceed with the definition of the numerical formulation used to find its solution. Here, we used a monolithic finite element method, as proposed in Colomés et al. (2023), Agarwal et al. (2024), where both the fluid velocity potential and the membrane deformation are solved through a unique coupled system. Before starting with the description of the numerical formulation, let us introduce some notation. We denote by $L^r(\Omega )$, $1\le r<\infty$, the spaces of functions such that their *r*-th power is absolutely integrable in $\Omega$. For the case in which $r=2$, we have a Hilbert space with an inner product

21 $$\begin{aligned} (u,v)_\Omega =\int _\Omega u(\textbf{x}) \, v(\textbf{x})d\Omega . \end{aligned}$$

and induced norm $\Vert u\Vert _{L^2(\Omega )}\equiv \Vert u\Vert _\Omega =(u,u)_\Omega ^{1/2}$. Abusing the notation, the same symbol as in (21) is used for the integral of the product of two functions, even if they are not in $L^2(\Omega )$, and for both the scalar and vector fields. The space of functions whose distributional derivatives up to order *m* are in $L^2(\Omega )$ are denoted by $H^m(\Omega )$. We considered the case of $m=1$, which is also a Hilbert space.

Let $\mathcal {V}= H^1(\Omega )$ be a functional space, $\mathcal {V}_{\Gamma _{\text{ fs }}}$ be the trace space of $\mathcal {V}$ on the free surface $\Gamma _{\text{ fs }}$, i.e., $\mathcal {V}_{\Gamma _{\text{ fs }}}=\{v|_{\Gamma _{\text{ fs }}}:v\in \mathcal {V}\}$, and $\mathcal {V}_{\Gamma _{\text{ m }}}$ be the trace space of $\mathcal {V}$ on the membrane $\Gamma _{\text{ m }}$. For a given set of parameters $[\tau , T_{\rho },m_\rho ]$, the parametric weak form of the problem reads: find $[\phi ,\eta ,\kappa ] \in \mathcal {V}\times \mathcal {V}_{\Gamma _{\text{ m }}}\times \mathcal {V}_{\Gamma _{\text{ fs }}}$ such that

22 $$\begin{aligned} &  B([\phi , \eta , \kappa ],[w, v, u], [\tau , T_{\rho }, m_{\rho }])=L([w, v, u]), \nonumber \\ &  \quad \quad \quad \forall [w,v,u] \in \mathcal {V} \times \mathcal {V}_{\Gamma _{m}} \times \mathcal {V}_{\Gamma _{fs}}, \end{aligned}$$

where the bilinear form is given by

23 $$\begin{aligned} \begin{aligned}&B([\phi , \eta , \kappa ], [w, v, u], [\tau , T_{\rho }, m_{\rho }])= (\nabla \phi , \nabla w)_{\Omega } \\&\qquad +\left( -i \omega \phi +g \kappa , \beta _h\left( u+\alpha _h w\right) \right) _{\Gamma _{f s}}+(i \omega \kappa , w)_{\Gamma _{f s}} \\&\qquad +\left( -m_\rho \omega ^2 \eta -i \omega \phi +g \eta , v\right) _{\Gamma _m} \\&\qquad +\left( T_\rho (1-i \omega \tau ) \nabla \eta , \nabla v\right) _{\Gamma _m}+(i \omega \eta , w)_{\Gamma _m}, \end{aligned} \end{aligned}$$

with the $\alpha _h$ and $\beta _h$ scaling parameters introduced for stability and dimensional consistency purposes, see (Colomés et al. 2023). The linear form reads

24 $$\begin{aligned} L([w, v, u])=\left( u_{\text{ in } }, w\right) _{\Gamma _{\text{ in } }}+\left( u_{\text{ out } }, w\right) _{\Gamma _{\text{ out } }}. \end{aligned}$$

We refer the reader to Agarwal et al. (2024) for a comprehensive description of the derivation of the weak problem (22).

Let us denote by $\Omega _h$ a conforming finite element triangulation of the domain $\Omega$; see Fig. 1b, from which we can construct conforming finite dimensional spaces for the velocity potential $\mathcal {V}_{h} \subset \mathcal {V}$, for the surface elevation at the free surface $\mathcal {V}_{\Gamma _{\text{ fs }},h}\subset \mathcal {V}_{\Gamma _{\text{ fs }}}$ and for the surface elevation at the membrane $\mathcal {V}_{\Gamma _{\text{ m }},h}\subset \mathcal {V}_{\Gamma _{\text{ m }}}$. Using this notation, the Galerkin FE formulation equivalent to (22) reads: find $[\phi _h,\eta _h,\kappa _h] \in \mathcal {V}_h\times \mathcal {V}_{\Gamma _{\text{ m }},h}\times \mathcal {V}_{\Gamma _{\text{ fs }},h}$ such that

25 $$\begin{aligned} \begin{aligned}&B([\phi _h, \eta _h, \kappa _h],[w_h, v_h, u_h], [\tau , T_{\rho }, m_{\rho }]) =L([w_h, v_h, u_h]) \\&\qquad \forall [w_h,v_h,u_h] \in \mathcal {V}_h \times \mathcal {V}_{\Gamma _{m},h} \times \mathcal {V}_{\Gamma _{fs},h}. \end{aligned} \end{aligned}$$

The variational spaces $\mathcal {V}_h$, $\mathcal {V}_{\Gamma _{\text{ fs }},h}$, and $\mathcal {V}_{\Gamma _{\text{ m }},h}$ are composed by complex-valued piecewise polynomials defined as

26 $$\begin{aligned} \mathcal {V}_h&=\left\{ w_h\in \mathcal {C}^0(\Omega _h):\ w_h\vert _K\in \mathbb {P}_r(K),\forall K\in \Omega _h\right\} , \end{aligned}$$

27 $$\begin{aligned} \mathcal {V}_{\Gamma _{\text{ fs }},h}&=\left\{ w_h\vert _E:\ w_h\in \mathcal {V}_h,\forall E\in \Gamma _{\text{ fs }}\right\} , \end{aligned}$$

28 $$\begin{aligned} \mathcal {V}_{\Gamma _{\text{ m }},h}&=\left\{ w_h\vert _E:\ w_h\in \mathcal {V}_h,\forall E\in \Gamma _{\text{ m }}\right\} , \end{aligned}$$

where $\mathbb {P}_r(K)$ is the space of Lagrange polynomials of degree $r\ge 1$ in an element *K*. In particular, in this work we use $r=2$.

Note that the problem in (22) is parameterized by $[\tau , T_{\rho }, m_{\rho }])=L([w_h, v_h, u_h]$, which can be arbitrary functions in both space and time. In this work, we are interested in investigating the behavior of floating viscoelastic membranes with time-invariant properties. Thus, we limited these variables to being only space dependent. In particular, we assumed that the parameters can be expanded by a linear combination of a finite set of orthogonal basis functions, $\{\psi _{\tau ,i}\}_{i=1}^{N_\tau }$, $\{\psi _{T,j}\}_{j=1}^{N_T}$ and $\{\psi _{m,k}\}_{k=1}^{N_m}$, such that

29 $$\begin{aligned}&\tau = \sum _{i=1}^{N_\tau }\psi _{\tau ,i}\tau _i,\qquad T_{\rho } = \sum _{j=1}^{N_T}\psi _{T,j}T_{\rho ,j}, \nonumber \\&\quad \qquad m_{\rho } = \sum _{k=1}^{N_m}\psi _{m,k}m_{\rho ,k}. \end{aligned}$$

Hereinafter, we denote $\boldsymbol{\tau }^{P}=[\tau _1,\ldots ,\tau _{N_\tau }]$, $\boldsymbol{T_{\rho }}^{P}=[T_{\rho ,1},\ldots ,T_{\rho ,N_T}]$, and $\boldsymbol{m_{\rho }}^{P}=[m_{\rho ,1},\ldots ,m_{\rho ,N_m}]$ as the vectors of values associated with each basis function for the damping coefficient $\tau$, the normalized tension $T_\rho$, and the submerged draft $m_\rho$, respectively. Then, the vector $\textbf{p} = [\boldsymbol{\tau }^{P}, \boldsymbol{T_{\rho }}^{P}, \boldsymbol{m_{\rho }}^{P}]^{T}$ contains the design parameters that will be tuned to find the optimal structural response; see Sect. 4. Note that the definition of the design variables as a linear combination of certain basis functions $\{\psi _{\tau ,i}\}_{i=1}^{N_\tau }$, $\{\psi _{T,j}\}_{j=1}^{N_T}$ and $\{\psi _{m,k}\}_{k=1}^{N_m}$ is a design choice that enables a general framework for space-varying coefficient optimization. Hence, the same formulation allows for homogeneous or highly varying properties in space. In Subsect. 4.3, we propose a particular choice of these basis functions.

Using the abovementioned notation, the final discrete problem can be rewritten as an algebraic system

30 $$\begin{aligned} A({\textbf {p}}) \boldsymbol{\theta } = {\textbf {b}}, \end{aligned}$$

where the matrix $A({\textbf {p}})$ contains the contributions from the bilinear form (23), and the vector $\boldsymbol{\theta } = [ \boldsymbol{\phi }^{P}, \boldsymbol{\eta }^{P}, \boldsymbol{\kappa }^{P} ]^{T}$ contains the vectors of degrees of freedom $\boldsymbol{\phi }^{P}$, $\boldsymbol{\eta }^{P}$, and $\boldsymbol{\kappa }^{P}$associated with the discrete fields $\phi _h$, $\eta _h$, and $\kappa _h$ respectively. The vector ${\textbf {b}}$ contains contributions of the linear form (24). Note that the matrix *A* in (30) is a function of the design variables; subsequently, the solution $\boldsymbol{\theta }$ will depend on the design parameters $\textbf{p}$.

### Wave generation and damping zones

The numerical wave field is generated within the domain using a Neumann boundary condition at the incoming boundary $\Gamma _{in}$, where the input wave velocity $u_{in}$ is prescribed based on the linear Airy wave theory, as prescribed in (3). To mitigate wave reflection from the floating membrane, a damping zone of length $L_d$ is introduced adjacent to the incoming boundary, as depicted in Fig. 1 where $L_d$ covers $\Omega _{d1}$. Inside this damping zone, an artificial wave damping function based on the $\phi _{n}-\eta$ type *Method 5* is applied to the free surface boundary, as outlined in Kim et al. (2014). This approach involves damping functions $\mu _1$ and $\mu _2$, where $k$ represents the wave number and $x_0$ is the starting point of the damping zone. The starting point of the damping zone, as can be seen from Fig. 1a, is the left inlet boundary $\Gamma _{in}$ where $x = 0$. These terms allow for selective absorption of waves reflected from the membrane by controlling the input wave elevation $\kappa _{in}$ and the input velocity potential $\phi _{in}$ along the damping zone, by prescribing them using the linear Airy wave theory. This exact method has been implemented in the same fashion in a previous study (Agarwal et al. 2024) that proved its effectiveness. Additional details about this method, including its ability to handle nonlinear and irregular waves, are discussed in Kim et al. (2014).

**Fig. 2:**
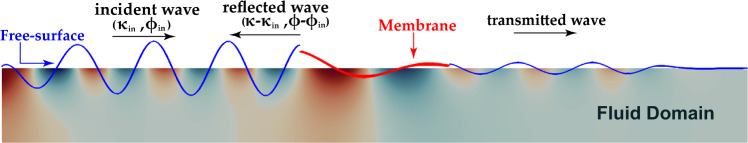
Illustration of the fluid domain with wave dynamics. The incoming wave ($\kappa _{\text {in}}, \phi _{\text {in}}$) is generated at the left boundary using a Neumann condition. Reflected waves ($\kappa - \kappa _{\text {in}}, \phi - \phi _{\text {in}}$) are measured left of the membrane and absorbed in a damping zone. Transmitted waves are damped at the right boundary using the Sommerfeld condition to prevent reflections. The free surface and membrane interactions demonstrate effective wave damping and energy transfer. The surface elevation $\kappa$ and membrane deformation $\eta$ are warped for visualization purposes

For outgoing waves, the Neumann boundary condition is also applied at the outgoing boundary $\Gamma _{out}$. We do so by prescribing $u_{out}$ for linear waves following the Sommerfeld radiation boundary equation as given by (33), with modifications involving an additional damping zone $\Omega _{d2}$ with length $L_{d}$ near the outgoing boundary to improve wave absorption in specific cases, as depicted in Fig. 1. This approach considering the damping of outgoing waves can be found in Agarwal et al. (2024) with further details. In Fig. 2 we depict the total concept of wave generation of the considered model.

31 $$\begin{aligned} &  \text {DFSC on } \Gamma _d: \quad \frac{\partial \phi }{\partial t} = -g \kappa - \mu _1 \left( \frac{\partial \phi }{\partial n} - \frac{\partial \phi _{\text {in}}}{\partial n} \right) \end{aligned}$$

32 $$\begin{aligned} &  \text {KFSC on } \Gamma _d: \quad \frac{\partial \eta }{\partial t} = \frac{\partial \phi }{\partial z} - \mu _2 (\kappa - \kappa _{\text {in}}) \end{aligned}$$

33 $$\begin{aligned} &  \text {where } \quad \mu _1 = \mu _0 \left( 1 - \sin \left( \frac{\pi }{2} \frac{x - x_0}{L_d} \right) \right) , \quad \text {and} \quad \mu _2 = k \mu _1 \nonumber \\ &  \nabla \phi \cdot \textbf{n} = i k \phi \quad \text {on } \Gamma _{\text {out}} \end{aligned}$$

## Optimization

In this section, we formulate the objective function and its approximation using the weak form. We derived the required gradient of the objective function with respect to the design parameters using the adjoint-based method.

### Objective function

From Agarwal et al. (2024) we find the absorbed power due to the viscoelastic membrane interaction with the fluid to be defined as

34 $$\begin{aligned} P_d(T_{\rho },\tau , \omega , \overline{\eta }({\textbf {p}})) = \frac{1}{2} \rho \omega ^2 \int _{\Gamma _m} T_{\rho } \tau | \nabla \overline{\eta } | ^2 d\Gamma _{m} . \end{aligned}$$

Now by substituting the approximated function of $\bar{\eta } \approx \eta _h$, where $\eta _h \in \mathcal {V}_{h}$, we obtained the approximated absorbed power of the membrane expressed as

35 $$\begin{aligned} P_d \approx \frac{1}{2} \rho \omega ^2 \int _{\Gamma _{m}} T_{\rho } \tau | \nabla \eta _h |^{2} d\Gamma _{m} . \end{aligned}$$

We want to maximize the absorbed power relative to the incoming power. Hence, we formulated our objective function as

36 $$\begin{aligned} q(\eta _{h}({\textbf {p}}), \omega ) = \frac{P_d}{P_{in}(\omega )} , \end{aligned}$$

where

37 $$\begin{aligned} P_{in}(\omega ) = \rho _w g \frac{\omega }{2k}\left( 1 + \frac{2kh}{\sinh (2kh)} \right) \frac{1}{2} \kappa _{in}^2 . \end{aligned}$$

Hereinafter, we will frequently refer to the objective function as the absorption coefficient.

### Adjoint-based optimization

To perform the optimization of the membrane, we made use of the so-called adjoint-based optimization (Errico 1997). Suppose we obtain the solution $\boldsymbol{\theta }$; thus, in this case, we obtain the solution from the discretized set of our governing equations. This solution is dependent on our design parameters **p**. Now, we need to compute the gradient of this objective function with respect to the design parameters. By applying the chain rule, we find that the gradient of *q* with respect to ${\textbf {p}}$ is defined as follows:

38 $$\begin{aligned} \frac{dq}{d{\textbf {p}}} = \frac{\partial q}{\partial {\textbf {p}}} + \frac{\partial q}{\partial \boldsymbol{\theta }}\cdot \frac{\partial \boldsymbol{\theta }}{\partial {\textbf {p}}} . \end{aligned}$$

We take the gradient of the objective function *q* with respect to a vector containing discrete design parameters. Hence, this gradient will be a vector with the length of the number of design parameters we are optimizing. Now, before diving into the adjoint-based method, we need to take a closer look at this gradient since the right-hand side of (38) is complex-valued. If we consider the real-valued function *q* to directly map the real-valued *n* design parameters such that

39 $$\begin{aligned} q({\textbf {p}}) : \mathbb {R}^n \rightarrow \mathbb {R} \end{aligned}$$

and for simplicity, use the definition of the total derivative of *q* with respect to one single design parameter $p_{0}$

40 $$\begin{aligned} \frac{dq}{dp}(p_0) = \lim _{h\rightarrow 0}{\frac{q(p_0 + h)-q(p_0)}{h}} , \end{aligned}$$

we find that by definition, this total derivative of *q* with respect to $p_{0}$ must be real-valued. Now, for any real-valued number *a*, it holds that

41 $$\begin{aligned} a = \Re (a) . \end{aligned}$$

Hence, we have proven that

42 $$\begin{aligned} \frac{dq}{d{\textbf {p}}} = \Re \left( \frac{\partial q}{\partial {\textbf {p}}} + \frac{\partial q}{\partial \boldsymbol{\theta }}\cdot \frac{\partial \boldsymbol{\theta }}{\partial {\textbf {p}}}\right) . \end{aligned}$$

Now, to work with our linear system of equations, as expressed in (30), we made use of the fact that

43 $$\begin{aligned} \frac{\partial }{\partial {\textbf {p}}}\left( A({\textbf {p}})\boldsymbol{\theta } \right) = \frac{\partial {\textbf {b}}}{\partial {\textbf {p}}} , \end{aligned}$$

where we find

44 $$\begin{aligned} A \frac{\partial \boldsymbol{\theta }}{\partial {\textbf {p}}} + \frac{\partial A}{\partial {\textbf {p}}} \boldsymbol{\theta } = {\textbf {0}} , \end{aligned}$$

and therefore

45 $$\begin{aligned} \frac{\partial \boldsymbol{\theta }}{\partial {\textbf {p}}} = A^{-1}\left( -\frac{\partial A}{\partial {\textbf {p}}} \boldsymbol{\theta } \right) . \end{aligned}$$

Now plugging (45) back in (38) we find our gradient to be expressed as

46 $$\begin{aligned} \frac{dq}{d {\textbf {p}}} =\frac{\partial q}{\partial {\textbf {p}}} + \frac{\partial q}{\partial \boldsymbol{\theta }} \cdot \left( A^{-1}\left( -\frac{\partial A}{\partial {\textbf {p}}} \boldsymbol{\theta } \right) \right) . \end{aligned}$$

Let us rewrite (46) such that we obtain the same result against a lower rate of computational work as described by Johnson (2006). Take the following term $\boldsymbol{\lambda }^{T} = \frac{\partial q}{\partial \boldsymbol{\theta }}A^{-1}$ which we found from the solution of the following so-called adjoint equation.

47 $$\begin{aligned} A^{\dagger }\boldsymbol{\lambda } = \frac{\partial q}{\partial \boldsymbol{\theta }} . \end{aligned}$$

Finally, we expressed the gradient as written in (38) such that we can implement it for our case where we are dealing with a linear system of equations.

48 $$\begin{aligned} \frac{dq}{d{\textbf {p}}} = \Re \left( \frac{\partial q}{\partial {\textbf {p}}} - \boldsymbol{\lambda }^T \frac{\partial A}{\partial {\textbf {p}}} \boldsymbol{\theta } \right) . \end{aligned}$$

There are two ways to find the partial derivatives present in (48). The first way is by using automatic differentiation packages that have the ability to compute partial derivatives rather efficiently and quickly. However, this effectiveness is diminished when the defined problem involves extensive use of third-party packages, as these can interfere with each other, making it often not possible for automatic differentiation packages to compute any gradients. The second way, which we implemented in this paper, is by hand. This approach can be error prone. Therefore, we need to demonstrate how these terms in (48) are derived. Subsequently, we used the finite difference method to validate the analytically derived gradient. The partial derivative of *q* with respect to the vector of the design parameters $\textbf{p}$ reads

49 $$\begin{aligned} \frac{\partial q}{\partial {\textbf {p}}} \equiv \begin{bmatrix} \frac{\partial q}{\partial \boldsymbol{\tau }^P}\\ \frac{\partial q}{\partial \boldsymbol{T_{\rho }}^P}\\ \frac{\partial q}{\partial \boldsymbol{m_{\rho }}^P} \end{bmatrix} = \frac{1}{P_{in}} \begin{bmatrix} \frac{1}{2} \rho \omega ^2 \int _{\Gamma _{m}} \frac{\partial \tau }{\partial \boldsymbol{\tau }^{P}} \, T_{\rho } \, | \nabla \eta _h |^{2} d\Gamma _{m}\\ \frac{1}{2} \rho \omega ^2 \int _{\Gamma _{m}} \tau \, \frac{\partial T_{\rho }}{\partial \boldsymbol{T_{\rho }}^{P}} \, | \nabla \eta _h |^{2} d\Gamma _{m}\\ \boldsymbol{0} \end{bmatrix} . \end{aligned}$$

To derive $\boldsymbol{\lambda }^T$ we in particular needed to find the partial derivative of *q* with respect to the solution $\boldsymbol{\theta }$. The adjoint of the matrix *A* can be found by plugging in the design parameters $\textbf{p}^{*}$ at which we are computing the gradient. We now took the partial derivative of *q* with respect to the degrees of freedom of the solution. Since $\eta _h$ is only a function of *x* in space, we obtained

50 $$\begin{aligned} \frac{\partial q}{\partial \boldsymbol{\theta }} \equiv \begin{bmatrix} \frac{\partial q}{\partial \boldsymbol{\phi }^P}\\ \frac{\partial q}{\partial \boldsymbol{\eta }^P}\\ \frac{\partial q}{\partial \boldsymbol{\kappa }^P} \end{bmatrix} = \frac{1}{P_{in}} \begin{bmatrix} \boldsymbol{0}\\ \rho \omega ^2 \int _{\Gamma _{m}} \, \tau \, T_{\rho } \, \frac{\partial \eta _h}{\partial x} \, \frac{\partial }{\partial \boldsymbol{\eta }^{P}}\left( \frac{\partial \eta _h}{\partial x}\right) d\Gamma _{m}\\ \boldsymbol{0} \end{bmatrix} . \end{aligned}$$

As mentioned before we can now express $\boldsymbol{\lambda }^T$ as

51 $$\begin{aligned} \boldsymbol{\lambda ^T} = \frac{\partial q}{\partial \boldsymbol{\theta }}A^{-1} . \end{aligned}$$

The term $\boldsymbol{\lambda }^{T}\frac{\partial A}{\partial {\textbf {p}}}\boldsymbol{\theta }$ can be expressed in the bilinear form. Hence, similar to (22) we can now write

52 $$\begin{aligned} \boldsymbol{\lambda }^T\frac{\partial A}{\partial {\textbf {p}}}(\textbf{p}^{*}) \boldsymbol{\theta } = \frac{\partial B}{\partial {\textbf {p}}}(\boldsymbol{\theta }, \boldsymbol{\lambda }, \textbf{p}^{*}) . \end{aligned}$$

This expression forms a horizontal vector, where each component is the partial derivative of matrix *A* with respect to each design parameter, represented by each element of $\textbf{p}$. Specifically, the derivative of matrix *A* acts as a bilinear operator on the vector $\boldsymbol{\lambda }^T$ from equation (51) and the solution $\boldsymbol{\theta }$. This operation computes a scalar value corresponding to each design parameter, resulting in a horizontal vector that matches the number of design parameters. The resulting vector structure is demonstrated in the following equation:

53 $$\begin{aligned} \begin{aligned} \frac{\partial B}{\partial {\textbf {p}}}&(\boldsymbol{\theta },\boldsymbol{\lambda },\textbf{p}^{*})=\left[ \begin{array}{ccc|ccc|ccc} \boldsymbol{\lambda }^T\frac{\partial A}{\partial \tau _{1}}\boldsymbol{\theta } & \cdots & \boldsymbol{\lambda }^T\frac{\partial A}{\partial \tau _{N_{\tau }}}\boldsymbol{\theta } & \boldsymbol{\lambda }^T\frac{\partial A}{\partial T_{\rho , 1}}\boldsymbol{\theta } & \cdots & \boldsymbol{\lambda }^T\frac{\partial A}{\partial T_{\rho , N_{T}}}\boldsymbol{\theta } & \boldsymbol{\lambda }^T\frac{\partial A}{\partial m_{\rho , 1}}\boldsymbol{\theta } & \cdots & \boldsymbol{\lambda }^T \frac{\partial A}{\partial m_{\rho , N_{m}}}\boldsymbol{\theta }\\ \end{array} \right] . \end{aligned} \end{aligned}$$

We note that the computational benefit of using this adjoint formulation is significant. Alternative gradient-based optimization approaches, such as a standard finite difference approach, would necessitate solving the finite element problem $N+1$ times (where $N$ is the number of design variables), the adjoint method computes the full gradient with only two system evaluations: one forward analysis to resolve the state variables (velocity potential $\phi$, membrane deflection $\eta$, and surface elevation $\kappa$) and one adjoint analysis to determine the sensitivity multipliers ($\lambda$), regardless of the dimension of the design space. This is specially relevant for the inhomogeneous cases considered in this study, where the number of design variables can be very high.

### Fourier series representation of the design variables

An alternative approach to significantly reduce the optimization time involves defining the design variables using Fourier series. Our formulation allows arbitrarily varying design variables in space. However, this method does not impose continuity requirements, which are beneficial in preventing discontinuities in material properties. By defining the design variables as truncated Fourier series, we inherently introduce continuity requirements. Additionally, this approach reduces the number of design parameters, as the spatial variation of the design variables is defined by the Fourier amplitudes. Consequently, the Fourier amplitudes become the design parameters that we optimize. A similar approach to optimize flapping kinematics in viscous flows has been carried out using adjoint-based optimization, and its effectiveness has been demonstrated (Van Schrojenstein Lantman and Fidkowski 2013). Therefore, we can define the Fourier amplitude design parameters as

54 $$\begin{aligned} \tau&= \sum _{n_{\tau }=0}^{K} \left( a_{n_{\tau }}^{\tau } \cdot \sin \left( \frac{n_{\tau } \pi x}{L} \right) + b_{n_{\tau }}^{\tau }\cdot \cos \left( \frac{n_{\tau } \pi x}{L} \right) \right) \end{aligned}$$

55 $$\begin{aligned} m_{\rho }&= \sum _{n_{m}=0}^{M} \left( a_{n_{m}}^{m_{\rho }} \cdot \sin \left( \frac{n_{m} \pi x}{L} \right) + b_{n_{m}}^{m_{\rho }}\cdot \cos \left( \frac{n_{m} \pi x}{L} \right) \right) . \end{aligned}$$

For each design variable, we constructed two vectors, each containing the amplitudes of the Fourier series corresponding to the sine and cosine. Hence, for the damping $\tau$, the design parameters are expressed as $\boldsymbol{\tau }^{P}=[\textbf{a}^{\tau }, \textbf{b}^{\tau }]^{T}$, where $\textbf{a}^{\tau } = [a_{0}^{\tau } \, a_{1}^{\tau } \cdots a_{K}^{\tau }]$ and $\textbf{b}^{\tau } = [b_{0}^{\tau } \, b_{1}^{\tau } \cdots b_{K}^{\tau }]$. The same can be applied for the mass $m_{\rho }$. Note that we considered a constant tension $T_{\rho }$ in space since we did not consider intermediate supports along the membrane. The design parameters can now be expressed as the following vector $\textbf{p}$:

56 $$\begin{aligned} {\textbf {p}} = \left[ \begin{array}{c} {\textbf {a}}^{\tau } \\ {\textbf {b}}^{\tau } \\ \hline T_{\rho } \\ \hline {\textbf {a}}^{m_{\rho }} \\ {\textbf {b}}^{m_{\rho }} \end{array} \right] \hspace{-2em} \begin{array}{c} \left. \begin{array}{c} \\ \\ \end{array} \right\} \boldsymbol{\tau ^{P}} \\ \\ \left. \begin{array}{c} \\ \\ \end{array} \right\} \boldsymbol{m_{\rho }^{P}} \end{array} . \end{aligned}$$

Using the same approach as demonstrated previously, we can find our adjoint-based gradient.

## Numerical results

The finite element model used in this study, along with adjoint-based optimization, was developed using the *Julia* programming language (Bezanson et al. 2017). For finite element analysis, the *Gridap* library (Verdugo and Badia 2021) was utilized, which is specifically tailored for high performance in the Julia Just-in-Time (JIT) compiler. This setup facilitates efficient computation for mixed-dimensional and mixed-order finite element formulations (Agarwal et al. 2024). The *Gridap* library enables users to manipulate and define the weak form of the equations, making it possible to integrate it with optimization libraries. Optimization procedures were implemented using the *NLopt* library (Johnson 2007), which supports local and global optimization techniques, with or without gradient information. The effective integration of these libraries allowed us to obtain the results of this study.

### Frequency-domain solution of the floating membrane

**Fig. 3:**
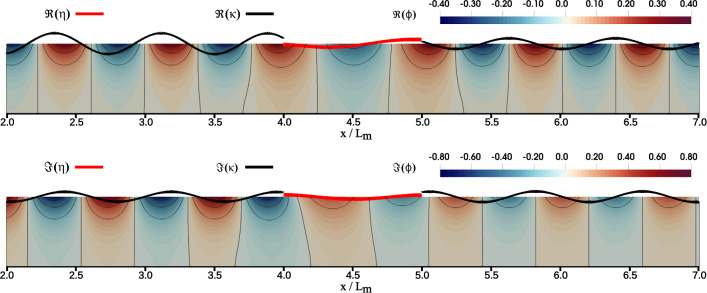
Visualization of the frequency-domain solution of the floating membrane depicting the complex-valued solutions of the unknown fields $\phi , \eta$, and $\kappa$. The red line depicts the deformation $\eta$ of the floating membrane, the black line depicts the free surface elevation of the surrounding fluid, and the blue-red colored areas depict the 2D field of the velocity potential $\phi$ (Agarwal et al. 2024). The surface elevation $\kappa$ and membrane deformation $\eta$ are warped for visualization purposes

In this study, we used the exact same domain with the corresponding discretization that satisfies the convergence requirements as done in Agarwal et al. (2024). Let us redefine the setup used where the finite element model is configured as follows: a two-dimensional vertical numerical domain with a uniform water depth of $h = 10$ meters. This domain extends over a total length of $L_{\Omega } = 33\,h$. The left boundary, $\Gamma _{in}$, facilitates the generation of incoming waves that excite the viscoelastic membrane, which measures $L_{m} = 2\,h = 20 \, \hbox {m}$ in length. Additionally, a damping zone has been implemented adjacent to $\Gamma _{in}$ to absorb the waves reflected by the floating membrane. This damping zone has a length of $L_{\Omega } = 15\,h$ and is in the range $x \in [-15\,h, \, 0]$. We used the artificial wave damping function described by the $\phi _n - \eta$ type Method 5 from Kim et al. (2014) and implemented in Agarwal et al. (2024). For the definition of these wave damping functions, we refer the reader to Kim et al. (2014) and Agarwal et al. (2024). The floating membrane is in the range $x \in [8\,h,\,10\,h]$. In contrast, the outgoing waves are mitigated by using the radiation boundary equation such that $u_{out}\,=\,ik\phi$ in (4). The computational domain employs second-order quadrilateral elements for discretization, with a fixed $\Delta x = 0.01\,h$. The vertical spacing $\Delta z$ changes exponentially across 20 elements, starting from $\Delta z = 0.0054h$ near the water surface and increasing to $\Delta z = 0.17h$ near the bottom. This arrangement is selected based on the convergence analysis as in Agarwal et al. (2024), considering factors such as structural response, wave length, and steepness of the shortest wave. Figure 3 shows an example of the solved problem in the frequency domain as extracted from Agarwal et al. (2024). As mentioned above, we deal with complex-valued fields for our solutions. By solving these complex-valued equations, we can determine the amplitudes and phases of the quantities for which we are solving.

### Behavior analysis of the objective function

Prior to advancing with any optimization strategies, we performed an analysis of the behavior of the objective function in two distinct scenarios. The viscoelastic membrane is subjected to a series of linear waves within a frequency spectrum of $\omega = [1.0, 8.0] \, \hbox {rad} \, s^{-1}$ and a wave amplitude $\kappa _{in}$ of $0.25 \, \hbox {m}$. We examined two scenarios. In both cases, the homogeneous material’s damping coefficient is maintained at $\tau = 0.05$. For the first scenario, the homogeneous mass is maintained at $m_{\rho }/L_m = 0.03$, and the simulation is performed throughout the frequency range with varying values of homogeneous tension $T_{\rho }$. In the second scenario, the homogeneous tension is kept at $T_{\rho }/gL_m^2 = 0.125$, and the simulation is rerun with varying homogeneous mass values $m_{\rho }$ for the same frequency range. It is important to note that in both cases $T_{\rho }$ and $m_{\rho }$ are considered homogeneous throughout the entire domain of the membrane.

#### Varying $T_{\rho }$

**Fig. 4:**
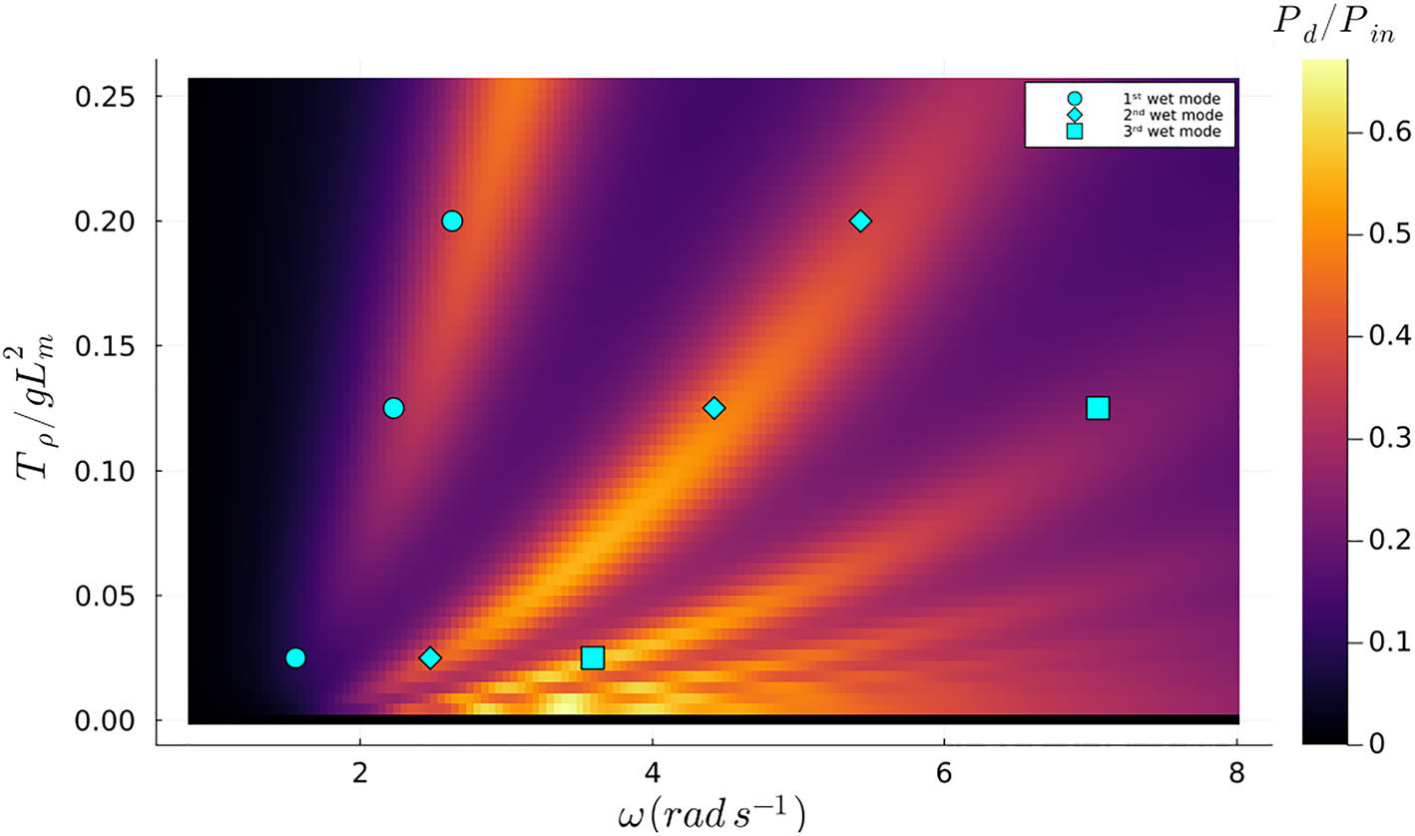
Contour plot of the objective function with fixed $\tau$ = 0.05 and fixed $m_{\rho }$ = 0.6, where the blue marks represent the samples from the first undamped three wet modes extracted from Agarwal et al. (2024)

Previous research has demonstrated that the ratio of $P_{d}/P_{in}$ exhibits local maxima at wet natural frequencies $\omega _{n} ^{w}$, confirming the significant relationship between these parameters and optimal energy absorption (Agarwal et al. 2024). This phenomenon is visually evident, as shown in Fig. 4.

**Fig. 5:**
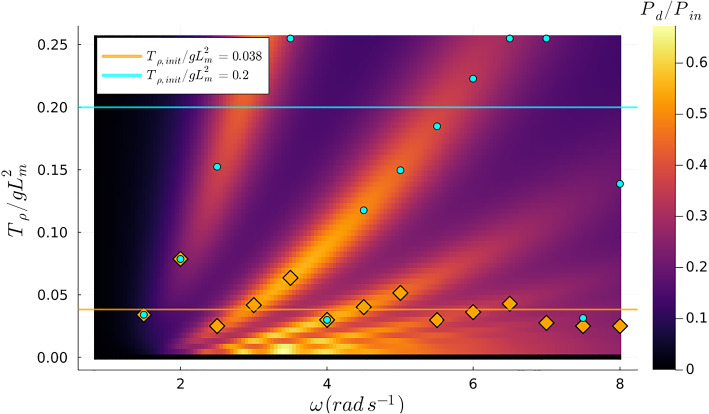
Contour plot of the objective function with the optimized values of $T_{\rho }$ using different initial values. Orange marks belong to the orange line and the blue marks belong to the blue line

Moreover, as observed in Fig. 4, the global maxima transition between different wet modes as the excitation frequency changes, suggesting that the global optimum can shift from one mode to another, depending on the proximity of the wet mode’s natural frequency to the exciter frequency. The choice of initial parameters for optimization is critical, as demonstrated in two sets of optimizations with different starting values of $T_{\rho }$. These optimizations were performed over a frequency range from $\omega = 1.0 \, \hbox {rad} \, s^{-1}$ to $\omega = 8.0 \, \hbox {rad} \, s^{-1}$ in steps of $\Delta \omega = 0.5 \, \hbox {rad} \, s^{-1}$, as shown in Fig. 5. At some frequencies, the optimizer converges to the upper limit of $T_{\rho }$, which is undesirable. However, it should be noted that all local optima align with the natural wet modes rather than the dry modes, underscoring the critical role of these modes in optimizing energy absorption. This insight aids in understanding the behavior of the absorption coefficient, even when the design parameters are modeled as inhomogeneous parameters.

#### Varying $m_{\rho }$

**Fig. 6:**
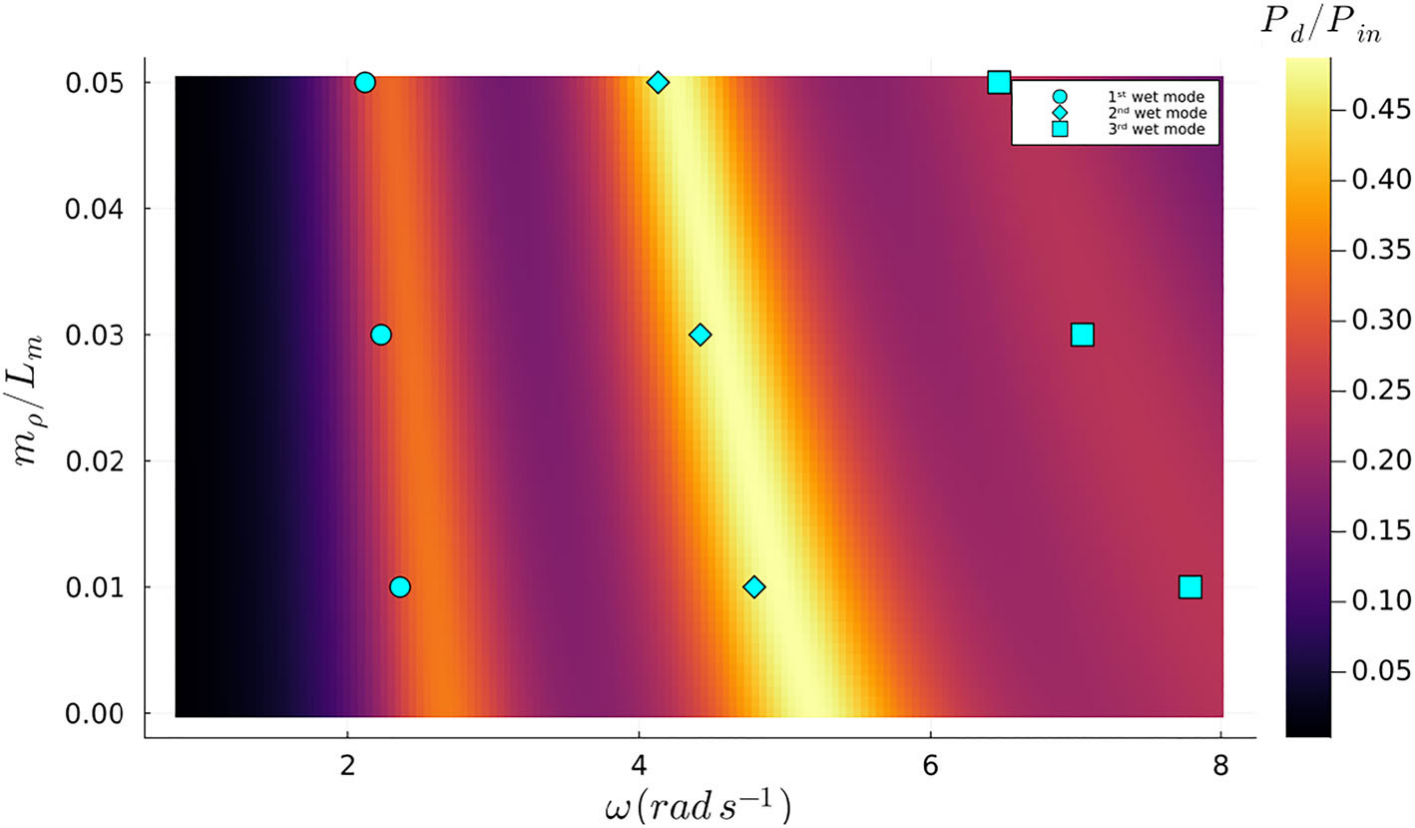
Contour plot of the objective function with fixed $\tau =0.05$ and fixed $T_{\rho }/gL_m^2=0.125$ where the blue marks are samples from the undamped first three wet modes as extracted from Agarwal et al. (2024)

**Fig. 7:**
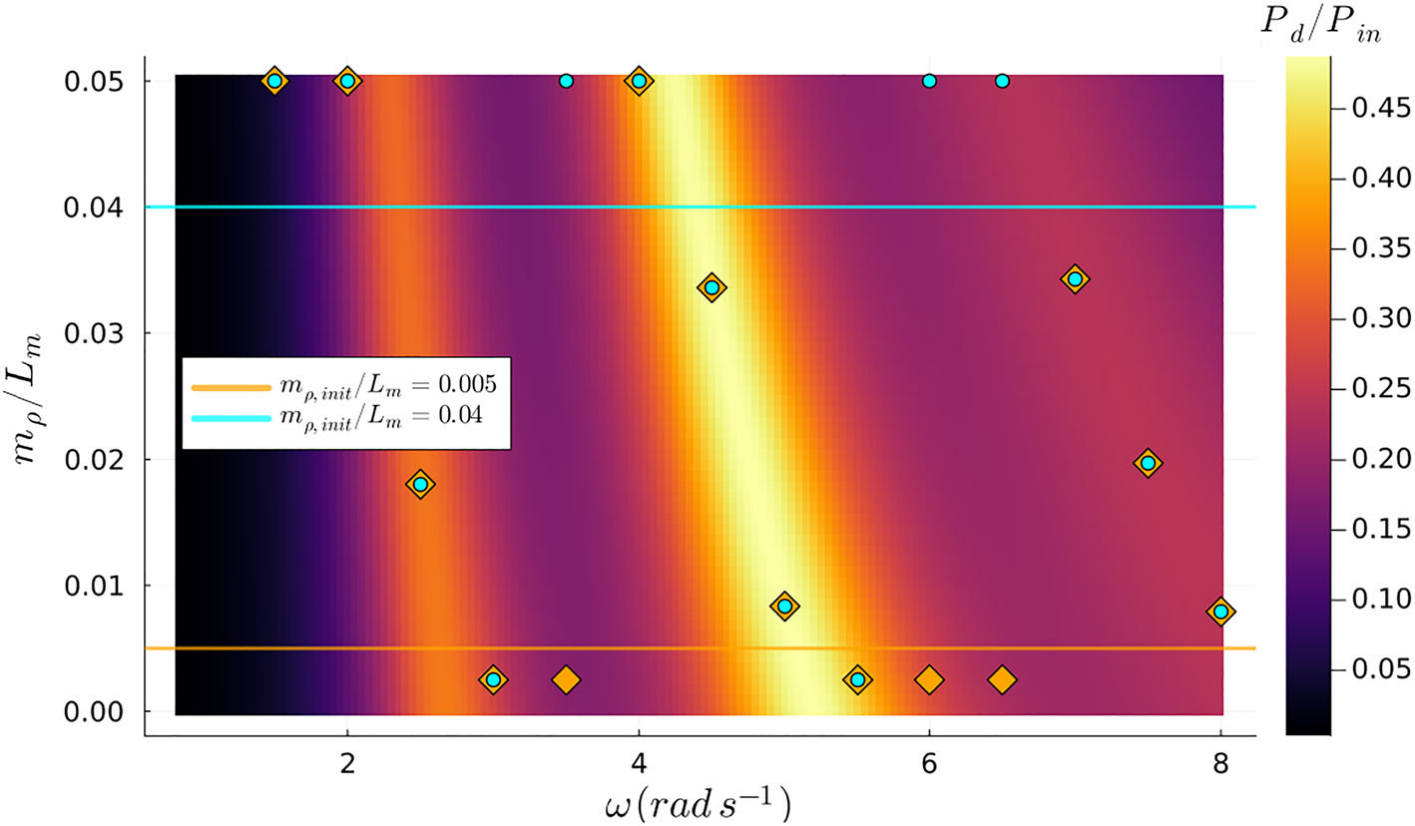
Contour plot of the objective function with the optimized values of $m_{\rho }$ using different initial values. Orange marks belong to the orange line and blue marks belong to the blue line

In this scenario, we set the tension at $T_{\rho }/gL_m^2 = 0.125$ and focused on optimizing the mass $m_{\rho }$ across the designated frequency range. Using a methodology analogous to that in the previous case, we utilized two different starting values for the optimization process. Similarly to what is observed in Fig. 4, the global maxima transition between different wet modes as the excitation frequency varies when the objective function only depends on the mass variable. This pattern is distinctly visible in Fig. 6. However, the relationship of the natural wet modes with respect to mass exhibits a different behavior compared to their interaction with tension. Specifically, the natural wet modes are triggered individually for particular excitation frequencies when the objective function is exclusively dependent on the mass. This contrasts to the behavior observed when the objective function was solely reliant on tension, where multiple natural wet modes could be activated within the same range of excitation frequencies. Nevertheless, even when focusing solely on optimizing the mass, we encountered the challenge of local optima due to the varying initial values, as demonstrated in Fig. 7. These observations are important because they provide a clearer understanding of the abrupt transitions in optimized design parameters attributed to the “switching” between natural wet modes depending on the excitation frequency.

### Optimizing $\tau$ and $m_{\rho }$

The next step was to simultaneously optimize both the damping $\tau$ and the mass $m_{\rho }$. For this purpose, we considered three scenarios, each characterized by a different fixed tension value, $T_{\rho }$, specifically $T_{\rho }/gL_m^2 = \{0.025, 0.125, 0.2\}$. From previous optimizations, it has been shown that the process is susceptible to yielding local optima influenced by the initial conditions provided to the optimizer. To mitigate this issue, we employed a global optimization approach to thoroughly explore the objective function space. In this research, we used the * multilevel single linkage* (MLSL) algorithm, a global optimizer that operates by initiating multiple local optimizations from various starting points in the objective function space (Kan et al. 1987; Johnson 2007). Accordingly, the use of a local optimizer is required for the sequence of local optimizations, for which the *low-storage BFGS* algorithm, commonly known as L-BFGS, has been selected (Luksan 2008). To enhance the efficiency of global optimization, we adopted a modified version of MLSL that utilizes a sequence of low-discrepancy starting points, rather than pseudo-random ones. This adjustment ensured a more uniform distribution of starting points within the objective function space, thus preventing clustering and improving the efficacy of the optimization process (Kucherenko and Sytsko 2005).

**Fig. 8:**
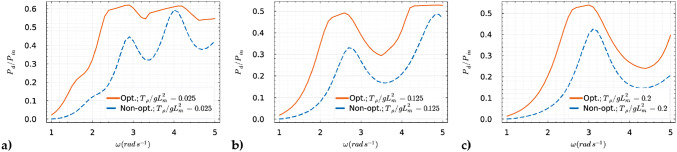
The global maxima of the objective function for the considered $T_{\rho }/gL_m^2 = \{0.025, 0.125, 0.2\}$ compared to its corresponding non-optimized naive choice of the mass and damping coefficient such that $m_{\rho }/L_m=0.03$ and $\tau =0.05$

**Fig. 9:**
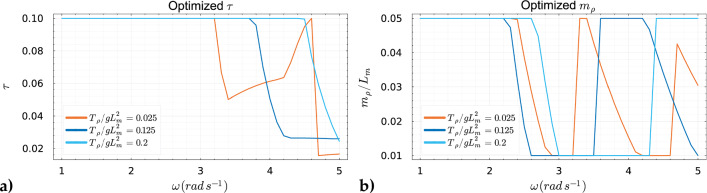
The damping coefficient $\tau$ and mass $m_{\rho }$ were simultaneously optimized across three distinct scenarios with varying tension values of $T_{\rho }/gL_m^2 = \{0.025, 0.125, 0.2\}$

As previously illustrated in Fig. 5 and Fig. 7, without employing a global optimizer, abrupt transitions occurred in our objective function when the excitation frequency was increased. These transitions represented a sudden shift from one local optimum to another, triggered solely by the choice of initial guesses. This behavior in the context of global optimization is undesirable. This issue has been mitigated by the implementation of a global optimizer, as discussed earlier. Using this approach, we observed from Fig. 8 that the global optima evolve more smoothly across the specified range of excitation frequencies. From the results, we observed that the membrane prefers a low pre-tension to reach optimum performance along a broad range of excitation frequency. This can be observed from Fig. 8a, where the optimized absorption coefficient experiences less variation in the higher frequencies, i.e., $2.6 \, \hbox {rad} \, s^{-1}\le \omega \le 5.0 \, \hbox {rad} \, s^{-1}$ compared to the two highest tension values considered. An explanation of this behavior can be found looking at Fig. 4, where we can see that for low pre-tension $T_{\rho }$ there are more optima of the objective function for the same considered range of excitation frequencies. We also see that these optima lie closer to each other compared to higher pre-tension values. Furthermore, if we study the returned optimized $\tau$ and $m_{\rho }$ as depicted in Fig. 9, we observe similar behavior as in Fig. 5 and Fig. 7 where we observe these sudden “jumps” indicating the “switching” between modes. As mentioned above, this so-called “switching” between the modes of the membrane is a consequence of the fact that the optimized properties of the membrane return a natural frequency corresponding to the excitation frequency. Finally, it should be noted the significant increase in the absorption coefficient when optimizing the mass and damping coefficient compared to the naive case where the design of the membrane properties is not optimized, as one can observe in Fig. 8. This indicates the potential for optimizing the viscoelastic membrane properties.

### Optimizing $\tau$, $T_{\rho }$, and $m_{\rho }$

**Fig. 10:**
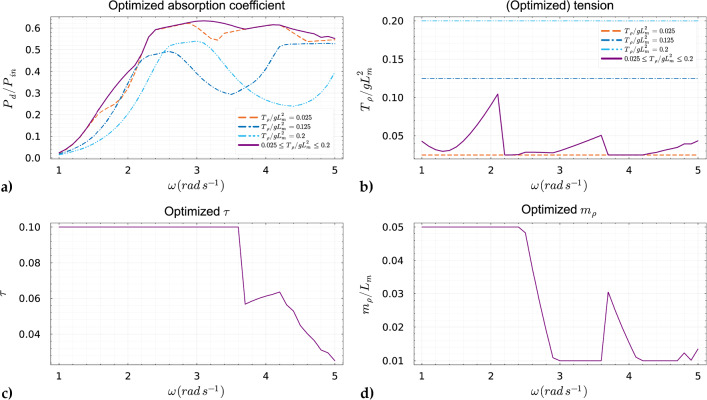
a) The maxima of the objective function across different excitation frequencies using the surface elevation $\kappa _{in}$ = 0.25 m. b) The resulting optimized $T_{\rho }$ compared with the non-optimized fixed $T_{\rho }$ cases. c) The resulting optimized $\tau$ when optimized with $T_{\rho }$ and $m_{\rho }$. d) The optimized $m_{\rho }$ when optimized with $T_{\rho }$ and $\tau$

**Fig. 11:**
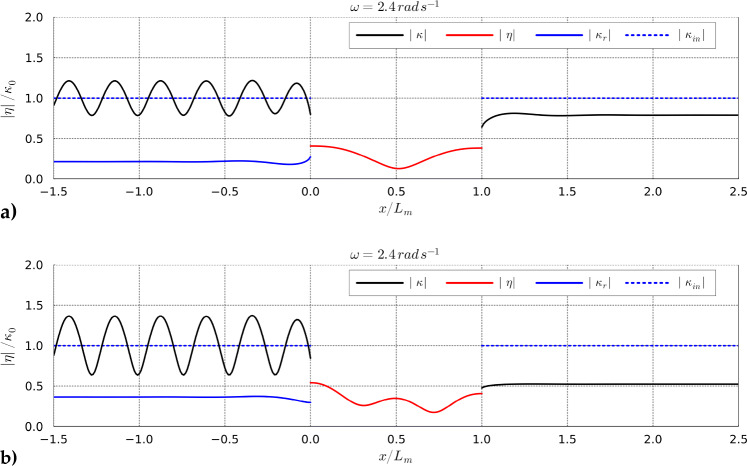
Amplitudes of the free surface elevation $\kappa$ and membrane deformation $\eta$. $\kappa _{r}$ depicts the reflected part of the incoming wave. $\kappa _{in}$ is the incoming wave excited from the inlet boundary with the wave amplitude $\kappa _{0}$. a) Frequency-domain solution of the naive case without optimization where $\{\tau , T_{\rho }/gL_m^2, m_{\rho }/L_m\} = \{0.05, 0.125, 0.03\}$. b) Frequency-domain solution with optimization where $\{\tau , T_{\rho }/gL_m^2, m_{\rho }/L_m\} = \{0.1, 0.025, 0.05\}$

In previous scenarios, we did not optimize the tension $T_{\rho }$. However, as observed in Fig. 9, the absorption coefficient achieves different maxima at various excitation frequencies, influenced by tension $T_{\rho }$. Therefore, optimizing tension $T_{\rho }$, mass $m_{\rho }$, and material damping $\tau$ simultaneously is a logical next step. This integrated approach allows us to fully capitalize on the optimization potential for homogeneous design variables in the membrane domain. In this scenario, we maintained the same lower and upper bounds for $m_{\rho }$ and $\tau$. However, since we are also optimizing for $T_{\rho }$, we have set a lower bound of $T_{\rho }/gL_m^2 = 0.025$ and an upper bound of $T_{\rho }/gL_m^2 = 0.2$. For this optimization, we used a global optimizer. The results of optimizing all three design parameters are depicted in Fig. 10. The optimization in this case clearly demonstrated its effectiveness by producing an optimized absorption coefficient without significant dips across the considered frequency range. The plateau of the absorption coefficient is notably broadened, highlighting the potential of optimizing all homogeneous design parameters to significantly reduce the likelihood of converging to local optima. Furthermore, Fig. 10b and Fig. 10d reveal distinct “jumps” in the optimized tension and mass, respectively. Referring to Fig. 5 and Fig. 7, these jumps likely indicate a “switching” of modes, ensuring that the properties of the floating membrane are adjusted so that its natural frequencies align closely with the excitation frequency, thus enhancing power absorption. Furthermore, Fig. 10c demonstrates that the optimal value of $\tau$ decreases as the excitation frequency increases. Finally, for visual purposes, we can see in Fig. 11b how optimization led to membrane deformation with a shape that exhibits more abrupt changes along the membrane space in contrast to Fig. 11a. From (34) we know that a larger absolute value of $\nabla \eta _{h}$ directly leads to a higher absorption coefficient. In both cases, we considered the same excitation frequency for the incoming wave, that is $\omega = 2.4 \, \hbox {rad} \, s^{-1}$.

### Optimizing $m_{\rho }(x)$

**Fig. 12:**
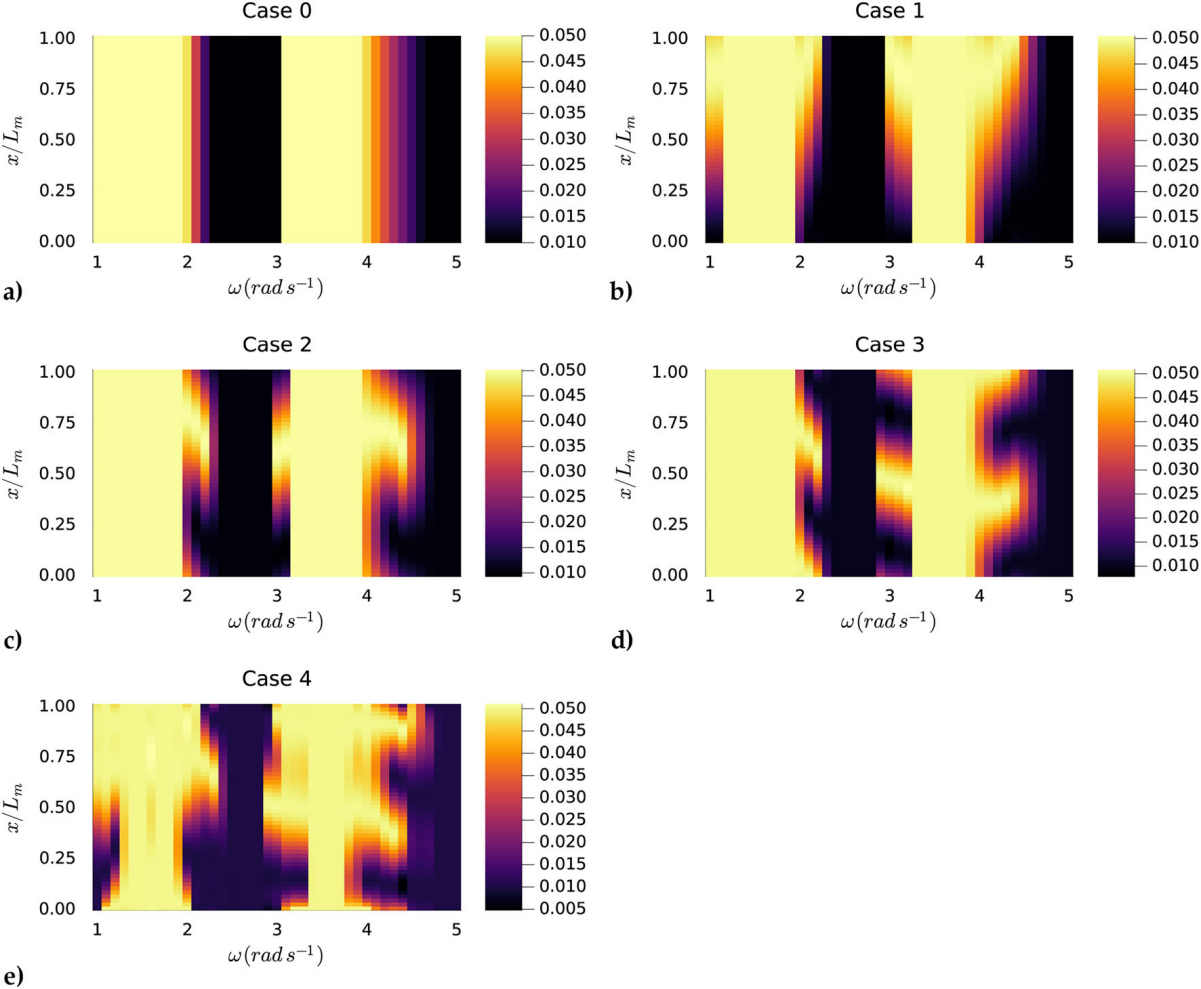
Contour plots displaying the effects of different summation terms on the modal mass $m_{\rho }(x)/L_{m}$ distribution across excitation frequencies $\omega$ using surface elevation $\kappa _{in} = 0.25$ m. Case 0: $n_{m}=0$. Case 1: $n_{m}=0, 1$. Case 2: $n_{m}=0, 2$. Case 3: $n_{m}=0, 3$. Case 4: $n_{m}=0$ to $n_{m}=3$. Each plot illustrates how the Fourier series representation of the mass varies with frequency for different term combinations

**Fig. 13:**
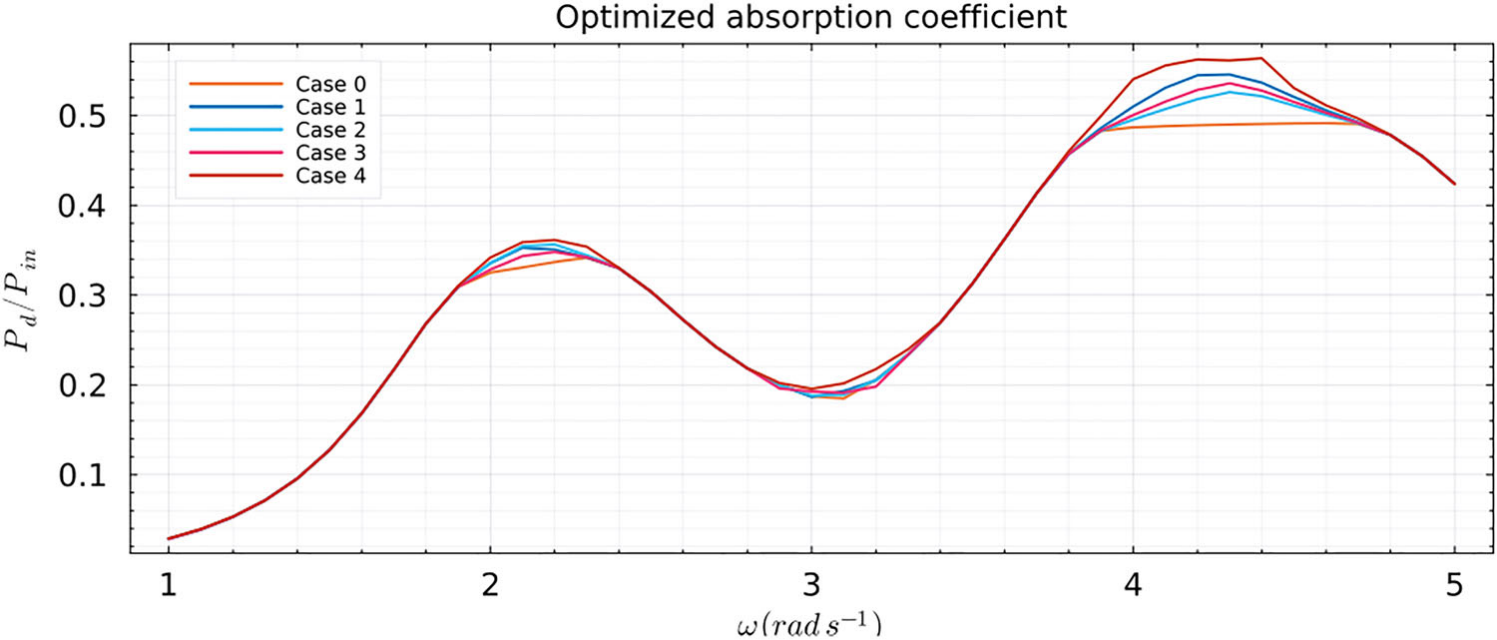
Optimized absorption coefficient for the five cases considered in Subsect. 5.5

For practical reasons, we only investigated the behavior of the optimized mass as a function of space along the membrane for separate frequencies. To study the behavior of the mass along the membrane, we used the definitions of the modal design parameters expressed in (54) and (55). For the damping, we only considered the case where $n_{\tau }=0$, and constrained it such that $\tau (x)=0.05$ resulting in constant values along the space of the membrane. Similarly for the tension, we considered a constant value along the space of the membrane which we did not optimize, such that $T_{\rho }/gL_m^2 = 0.125$. Hence, to solely understand the behavior of the mass, we did not optimize the damping $\tau$ or the tension $T_{\rho }$. For the mass, we considered several cases with different assemblies of the Fourier series. The first case we considered only the summation of the $n_{m}=0$ and $n_{m}=1$ Fourier amplitudes. The second case we considered only the summation of the $n_{m}=0$ and $n_{m}=2$ Fourier amplitudes. The third case we considered only the summation of the $n_{m}=0$ and $n_{m}=3$ Fourier amplitudes. We then considered the summation of the Fourier amplitudes $n_{m}=0$ to $n_{m}=3$, which is the fourth case. In addition, we compared these cases to a zeroth case where we only considered $n_{m} = 0$, i.e., we optimized for homogeneous mass along the space of the membrane. In all cases, we bounded the mass such that $0.01 \le m_{\rho }(x)/L_{m} \le 0.05$. Before analyzing the optimized mass profiles, we looked at the optimized absorption coefficient. Figure 13 shows an optimized absorption coefficient with two clear peaks representing the two wet modes of the floating viscoelastic membrane. Notably, the second wet mode absorbs the most energy in all cases. Case 4 generally returns a mass profile where the membrane absorbs the most energy compared to the other modes, followed by Case 1, suggesting that $n_{m}=0$ and $n_{m}=1$ are the most dominant contributions in the mass profile for energy absorption. Figure 12 shows that in all cases, the optimizer returns the predefined lower bound when the membrane is excited by frequencies outside the natural frequencies of the wet modes. This suggests that when excited by such frequencies, the best design strategy is to minimize the mass to reduce inertia, allowing the membrane to oscillate more. Case 1 shows a clear pattern where the wet modes of the floating membrane are activated as much as possible by introducing a light upstream part and increasing the mass downstream. As we continued to analyze the behavior of the optimized mass profiles in Case 2 and Case 3, it became evident that the optimizer struggled to fully replicate the desired mass distribution pattern due to the limitations imposed by the specific Fourier series combinations. The restriction to certain Fourier amplitudes constrained the optimizer’s ability to create an ideal mass profile that effectively responds to varying excitation frequencies. In Case 4, however, the inclusion of a wider range of Fourier amplitudes ($n_{m}=0$ to $n_{m}=3$) allowed for a more flexible and efficient mass distribution. This resulted in a mass profile that maximizes energy absorption by adapting to the incoming wave frequencies more effectively. The light upstream reduced early wave reflection, while the heavier tail absorbed the lower amplitude waves, thus broadening the spectrum of wet modes. This adaptive strategy is critical for optimizing membrane performance. By adjusting the mass along the membrane, the optimizer can enhance the interaction between the membrane and the waves, leading to better overall energy absorption. Although we successfully optimized for a homogeneous mass distribution in Case 0, it is important to note that this case performs the worst in terms of achieving a high optimized absorption coefficient. This further emphasizes that increasing the number of degrees of freedom in the optimization process expands the search space, leading to improved optimized properties for the viscoelastic membrane. This principle is consistent with the findings in Agarwal et al. (2024), where it was noted that optimizing the mass distribution can significantly impact the membrane’s ability to handle varying wave frequencies. Moreover, it is important to highlight a limitation encountered in Case 4. The optimizer failed to respect the predefined lower bound for the mass, indicating a limitation of the optimization library used.

### Optimizing a 2D floating membrane around a monopile

**Fig. 14:**
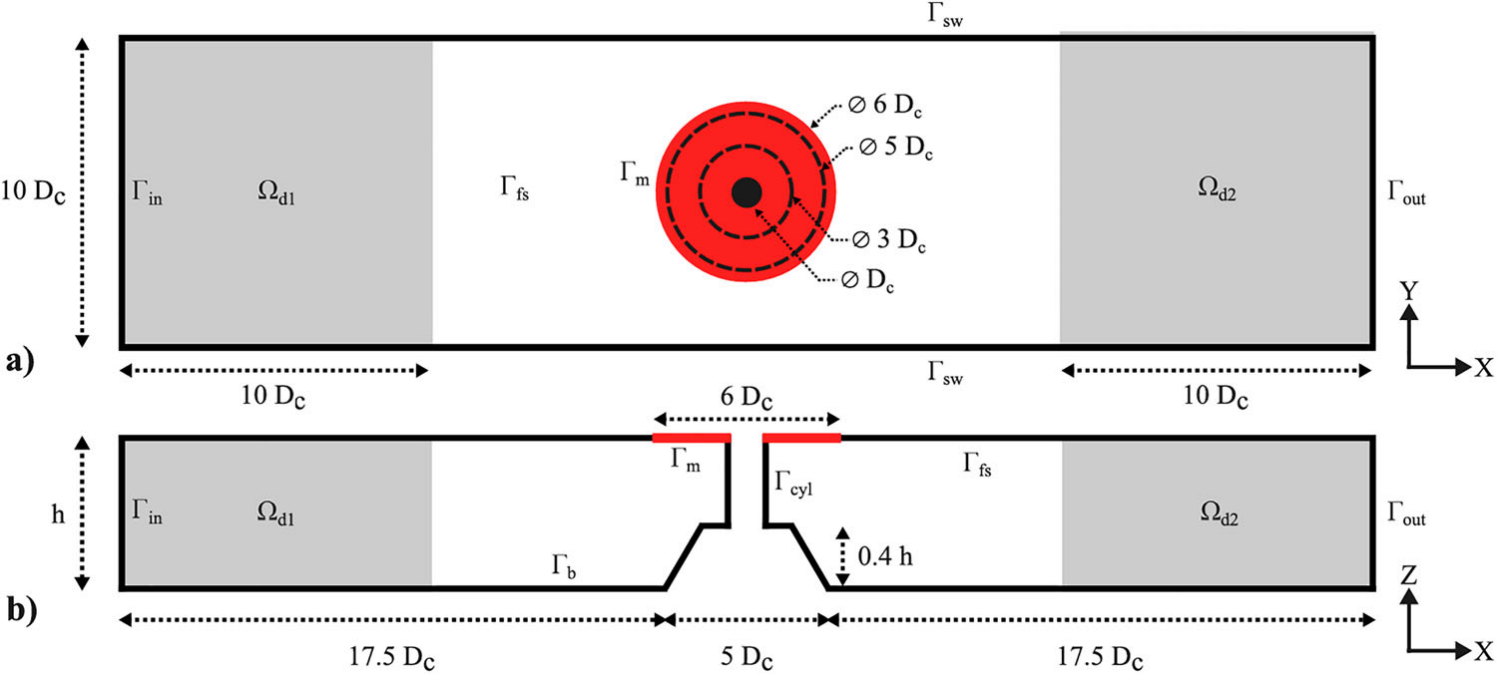
Diagram of the numerical domain featuring a monopile with boundary $\Gamma _{c_f}$, encircled by a viscoelastic floating membrane $\Gamma _m$ (indicated in red). The monopile is mounted on a submerged conical island (Agarwal et al. 2024). **a** Top view of the domain. **b** Cross-sectional view along the middle plane of the domain

In this section, we demonstrate the practical application of the proposed methodology by proving its inherent ability to optimize for complexer situations, including a non-trivial bathymetry and non-trivial membrane geometry. We considered the same setup as presented in a previous study (Agarwal et al. 2024), inspired by the dimensions of wind-turbine monopile foundations at the North Hoyle wind farm, located off the coast of North Wales. These monopiles typically have a diameter of $4 \, \textrm{m}$ and are installed at depths ranging between $7$ and $11 \, \textrm{m}$. In our case, we modeled a monopile with diameter $D_c = 4 \, \textrm{m}$ and length $L_c = 1.5 D_c$, placed in the center of a numerical domain with width $W_\Omega /D_c = 10$, length $L_\Omega /D_c = 40$, and depth $h = 10 \, \textrm{m}$. The monopile rests on a submerged conical island with a top diameter of $3D_c$, bottom diameter $5D_c$, and height $0.4h$. Surrounding the monopile is a viscoelastic floating membrane $\Gamma _m$ of diameter $D_m = 6D_c$. Similar to the previous cases, the membrane’s mass, pre-tension, and damping coefficient are optimized in this study to maximize the objective function. This setup serves as a representative case to evaluate the performance of our optimization framework for viscoelastic membranes in realistic environments (see Fig. 14).

**Fig. 15:**
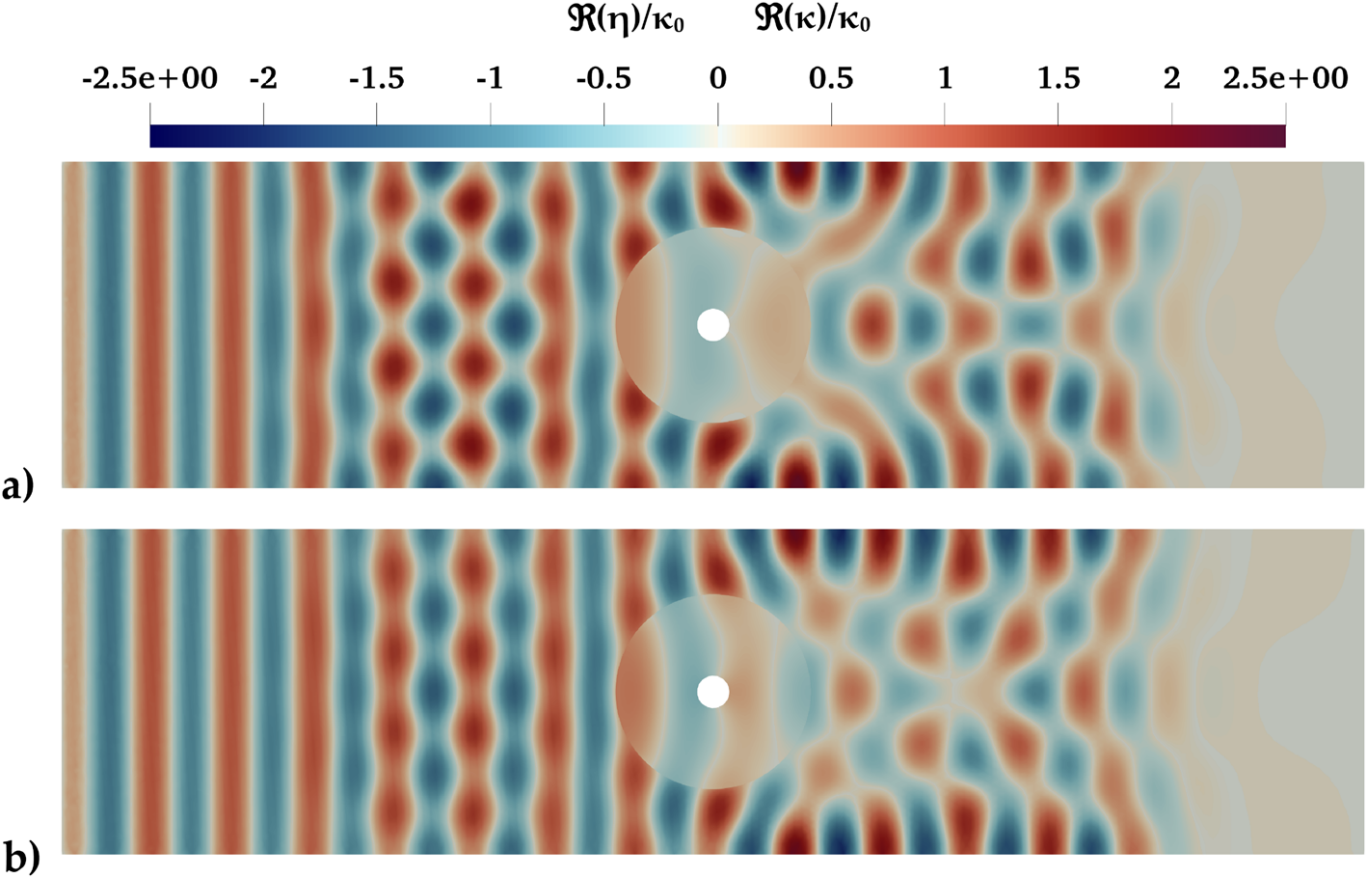
Comparison of the simulated free surface and membrane deformation between the naive case (a) extracted from Agarwal et al. (2024) and the optimized case (b). The plotted values represent the real parts of the normalized deformation, $\Re (\eta ) / \kappa _0$ and $\Re (\kappa ) / \kappa _0$, where $\kappa _0$ is the incoming wave height. The optimized case exhibits a larger wavelength (approximately 1.5) compared to the naive case (approximately 1.25), leading to a larger absolute gradient of $\nabla \eta _h$ and improved power absorption

We can formulate the case where the viscoelastic membrane surrounding the monopile is characterized by an inhomogeneous mass and damping distribution, while the tension is considered homogeneous. The mass and damping distribution are modeled using a Fourier series expansion in polar coordinates, which allows the representation of spatial variations, which are expressed as follows:

57 $$\begin{aligned} m_\rho (r, \theta )= &  \sum _{n=0}^{N_{r}^{m_\rho }-1} \sum _{k=-K_{\theta }^{m_\rho }}^{K_{\theta }^{m_\rho }} \left( A_{n,k}^{m_\rho } \sin (k\theta )\right. \nonumber \\ &  \left. + B_{n,k}^{m_\rho } \cos (k\theta ) \right) \left( \frac{r}{D_m / 2} \right) ^n, \end{aligned}$$

58 $$\begin{aligned} \tau (r, \theta )= &  \sum _{n=0}^{N_{r}^\tau -1} \sum _{k=-K_{\theta }^\tau }^{K_{\theta }^\tau } \left( A_{n,k}^\tau \sin (k\theta )\right. \nonumber \\ &  \left. + B_{n,k}^\tau \cos (k\theta ) \right) \left( \frac{r}{D_m / 2} \right) ^n, \end{aligned}$$

where $A_{n,k}$ and $B_{n,k}$ are the Fourier coefficients, $r$ and $\theta$ are the radial and angular coordinates, $N_r$ and $K_\theta$ are the numbers of radial and angular modes, and $L_m$ is a reference length scale (the radius of the membrane). This approach ensures flexibility in capturing spatial variations of the mass and damping coefficient while maintaining numerical efficiency. Thus, the final design vector is given by

59 $$\begin{aligned} {\textbf {p}} = \left[ \begin{array}{c} {\textbf {A}}^{\tau } \\ {\textbf {B}}^{\tau } \\ \hline T_{\rho } \\ \hline {\textbf {A}}^{m_{\rho }} \\ {\textbf {B}}^{m_{\rho }} \end{array} \right] \hspace{-2em} \begin{array}{c} \left. \begin{array}{c} \\ \\ \end{array} \right\} \boldsymbol{\tau ^{P}} \\ \\ \left. \begin{array}{c} \\ \\ \end{array} \right\} \boldsymbol{m_{\rho }^{P}} \end{array} . \end{aligned}$$

Here, $T_{\rho }$ is a scalar because we considered it to be homogeneous, while $\textbf{A}$ and $\textbf{B}$ are arrays of coefficients whose size depends on the chosen number of radial and angular modes.

We considered a simulation case where we excite the structure with an incoming wave having a frequency $\omega = 2.5 \, \hbox {rad} \, s^{-1}$. Moreover, we set the following bounds: $1.0 \times 10^-2 \le \tau (\textbf{x}) \le 1.0 \times 10^-1$, $3.54 \times 10^-3 \le T_{\rho }/g D_{m}^{2} \le 8.85 \times 10^-2$ & $2.4 \times 10^-2 \le m_{\rho }(\textbf{x})/D_{m} \le 2.4 \times 10^-1$. To ensure that the optimization remains manageable and avoids an excessive number of optimization parameters, we opted to make only the mass inhomogeneous, setting $N_{r}^{m_{\rho }} = 2$ and $K_{\theta }^{m_{\rho }} = 4$. For damping, we chose to assume homogeneous damping, even though our approach can accommodate inhomogeneous damping along the membrane’s span. Similarly to a previous study, we considered a real potential material for the construction of a viscoelastic membranes, namely high-density polyethylene (HDPE) with a density of $\rho _{m} = 970 \, \text {kg m}^{-3}$ (Agarwal et al. 2024). Therefore, the optimized mass can be practically realized by thickening or thinning the membrane.

The optimization returned a membrane that should have a damping coefficient $\tau = 0.10$, $T_{\rho }/g D_{m}^2 = 4.27 \times 10^-3$ & $m_{\rho }(\textbf{x})/D_{m} = 2.4 \times 10^{-1}$. It is worth noting that, despite allowing for an inhomogeneous mass, the optimizer returned a homogeneous mass. This behavior was also observed in the 2D case, as shown in Fig. 12, where the optimizer selected the lower or upper bound for the mass at certain frequencies. The maximum of the objective function found is circa 0.44. For reference, we compared the optimized case with the non-optimized case found in Agarwal et al. (2024) and found a $17 \%$ increase in power absorption. Additionally, we compared the returned simulations of the optimized and non-optimized cases using *ParaView* as can be seen in Fig. 15. In the optimized case, we observe a propagated deformation wave with a wavelength of approximately 1.5. In contrast, the naive case exhibits a shorter propagated wave with a wavelength of around 1.25. This behavior aligns with the phenomenon described in the 2D scenario shown in Fig. 11. As a result, the optimized case produces a larger absolute value of $\nabla \eta _{h}$, which directly increases the absorption coefficient, as can be derived from the definition of the objective function.

## Conclusion

This study presented an adjoint-based, PDE-constrained optimization framework to maximize the power absorption of viscoelastic floating membranes. We formulated a unified formulation for the assessment of inhomogeneous floating membranes and we investigated the influence of both homogeneous and inhomogeneous material properties on energy harvesting efficiency across a broad frequency range.

The results demonstrate that tuning the tension and mass of the membrane allows for an alignment of the wet natural frequencies of the system with the excitation wave. Specifically, membranes with lower pre-tension exhibited higher adaptability, achieving high absorption coefficients across a wider bandwidth. Furthermore, the optimization of inhomogeneous mass distributions significantly outperformed homogeneous designs. During the optimization process, higher-order wet modes are activated by redistributing mass along the membrane length, enhancing the coupling effects between the structure and the fluid. These findings were also observed in a realistic 3D application involving a membrane surrounding a monopile, where optimization yielded a 17% increase in power absorption compared to a standard design.

While the numerical results highlight the potential of adaptive flexible structures, future work must address the translation to physical experiments. Experimental uncertainties, such as manufacturing tolerances in membrane thickness or tension, could lead to deviations in the actual wet natural frequencies, potentially degrading the performance of designs optimized for a single sharp resonance. Future studies should therefore consider robust optimization strategies to ensure performance stability under such parameter uncertainties. Overall, this research confirms that flexible, adaptive design configurations are essential for the next generation of wave energy converters and floating breakwaters.

## Data Availability

The authors will make the data available upon reasonable request.

## Declarations

### Replication of results

The results of the manuscript have been obtained using a monolithic Finite Element library for the simulation of very large floating structures, see (Colomes yyy). Further details of the optimization software are detailed in Sect. 5. The authors will make the scripts used to generate the results available upon reasonable request.

## References

[CR1] Agarwal S, Colomes Gene O, Metrikine A (2024) Dynamic analysis of viscoelastic floating membranes using monolithic finite element method. J Fluids Struct 129:104167. 10.1016/j.jfluidstructs.2024.104167

[CR2] Bezanson J, Edelman A, Karpinski S, Shah VB (2017) Julia: a fresh approach to numerical computing. SIAM Rev 59(1):65–98. 10.1137/141000671

[CR3] Boren B (2021) Distributed embedded energy converters for ocean wave energy harvesting: enabling a domain of transformative technologies: preprint. Renew Energy 9

[CR4] Collins I, Hossain M, Dettmer W, Masters I (2021) Flexible membrane structures for wave energy harvesting: a review of the developments, materials and computational modelling approaches. Renew Sustain Energy Rev 151:111478. 10.1016/j.rser.2021.111478

[CR5] Colomes O. MonolithicFEMVLFS.jl. https://github.com/oriolcg/MonolithicFEMVLFS.jl. 10.4121/19601419

[CR6] Colomés O, Verdugo F, Akkerman I (2023) A monolithic finite element formulation for the hydroelastic analysis of very large floating structures. Int J Numer Meth Eng 124(3):714–751. 10.1002/nme.7140

[CR7] Drew B, Plummer AR, Sahinkaya MN (2009) A review of wave energy converter technology. Proc Inst Mech Eng Part A J Power Energy 223(8):887–902. 10.1243/09576509JPE782

[CR8] Errico RM (1997) What is an adjoint model? Bull Am Meteor Soc 78(11):2577–2592

[CR9] Holkema KJ, Aalbers C, Wellens PR (2023) Force and water jet impact reduction on adjacent structures by means of free surface breakwaters. Int Shipbuild Prog. 10.3233/isp-230012

[CR10] Johnson SG (2006) Notes on Adjoint Methods for 18.335. https://math.mit.edu/~stevenj/18.336/adjoint.pdf

[CR11] Johnson SG (2007) The NLopt nonlinear-optimization package. https://github.com/stevengj/nlopt

[CR12] Khabakhpasheva TI, Korobkin AA (2002) Hydroelastic behaviour of compound floating plate in waves. J Eng Math 44(1):21–40. 10.1023/A:1020592414338

[CR13] Kim MW, Koo W, Hong SY (2014) Numerical analysis of various artificial damping schemes in a three-dimensional numerical wave tank. Ocean Eng 75:165–173. 10.1016/j.oceaneng.2013.10.012

[CR14] Koley S, Trivedi K, Vipin V (2022) Hydroelastic analysis of floating long viscoelastic plate in shallow water. Mater Today Proc 49:2234–2238. 10.1016/j.matpr.2021.09.334

[CR15] Kucherenko S, Sytsko Y (2005) Application of deterministic low-discrepancy sequences in global optimization. Comput Optim Appl 30(3):297–318. 10.1007/s10589-005-4615-1

[CR16] Li X, Xiao Q (2022) A numerical study on an oscillating water column wave energy converter with hyper-elastic material. Energies 15(22):8345. 10.3390/en15228345

[CR17] López I, Andreu J, Salvador C, Alegría IM, Kortabarria I (2013) Review of wave energy technologies and the necessary power-equipment. Renew Sustain Energy Rev 27:413–434. 10.1016/j.rser.2013.07.009

[CR18] Lu C, Xu L, Guo A, Liu J (2025) Shape optimization of floating bridge pontoons with mooring constraints under wave actions. Struct Multidiscip Optim 68(3):1–19

[CR19] Luksan L (2008) Limited-memory projective variable metric methods for unconstrained minimization. Technical report, Institute of Computer Science, Pod Vodarenskou Vezi 2, 18207 Praha 8

[CR20] Meylan MH, Bennetts LG, Peter MA (2017) Water-wave scattering and energy dissipation by a floating porous elastic plate in three dimensions. Wave Motion 70:240–250. 10.1016/j.wavemoti.2016.06.014

[CR21] Michele S, Buriani F, Renzi E, Rooij M, Jayawardhana B, Vakis AI (2020) Wave energy extraction by flexible floaters. Energies 13(23):6167. 10.3390/en13236167

[CR22] Qin H, Su H, Wen Z, Liang H (2025) Latching control of a point absorber wave energy converter in irregular wave environments coupling computational fluid dynamics and deep reinforcement learning. Appl Energy 396:126282. 10.1016/j.apenergy.2025.126282

[CR23] Renzi E (2016) Hydroelectromechanical modelling of a piezoelectric wave energy converter. Proc Royal Soc A Math Phys Eng Sci 472(2195):20160715. 10.1098/rspa.2016.0715

[CR24] Rinnooy Kan AHG, Rinnooy KAHG, Timmer GT (1987) Stochastic global optimization methods. Part 1: clustering methods. Math Program 39(1):27–56. 10.1007/bf02592070

[CR25] Shadmani A, Nikoo MR, Etri T, Gandomi AH (2023) A multi-objective approach for location and layout optimization of wave energy converters. Appl Energy 347:121397. 10.1016/j.apenergy.2023.121397

[CR26] Solano D, Sarojini D, Rajaram D, Mavris DN (2022) Adjoint-based analysis and optimization of beam-like structures subjected to dynamic loads. Struct Multidiscip Optim 65(2):52

[CR27] Sree DK, Mandal S, Law AWK (2021) Surface wave interactions with submerged horizontal viscoelastic sheets. Appl Ocean Res 107:102483. 10.1016/j.apor.2020.102483

[CR28] Trivedi K, Koley S (2022) Modeling the viscoelasticity of floating membrane in water waves. Mater Today Proc. 10.1016/j.matpr.2022.07.047

[CR29] Van Schrojenstein Lantman M, Fidkowski K (2013) Adjoint-based optimization of flapping kinematics in viscous flows. In: 21st AIAA computational fluid dynamics conference, p. 15. American institute of aeronautics and astronautics, San Diego, CA. 10.2514/6.2013-2848

[CR30] Verdugo F, Badia S (2021) The software design of Gridap: a Finite Element package based on the Julia JIT compiler. Comput Phys Commun. 10.1016/j.cpc.2022.108341

[CR31] Wang J, Wang X (2023) Ant colony optimization based array design of wave energy converters. 2023 5th Asia Energy and Electrical Engineering Symposium (AEEES). 10.1109/aeees56888.2023.10114112

[CR32] Zhang H et al (2021) Optimization of a three-dimensional hybrid system combining a floating breakwater and a wave energy converter array. Energy Convers Manage 247:114717. 10.1016/j.enconman.2021.114717

[CR33] Zhang M, Schreier S (2022) Review of wave interaction with continuous flexible floating structures. Ocean Eng 264:112404. 10.1016/j.oceaneng.2022.112404

